# Comprehensive geriatric assessments in integrated care programs for older people living at home: A scoping review

**DOI:** 10.1111/hsc.12793

**Published:** 2019-06-21

**Authors:** Annerieke Stoop, Manon Lette, Paul F. van Gils, Giel Nijpels, Caroline A. Baan, Simone R. de Bruin

**Affiliations:** ^1^ Centre for Nutrition Prevention and Health Services National Institute for Public Health and the Environment Bilthoven the Netherlands; ^2^ Amsterdam Public Health Research Institute, Department of General Practice and Elderly Care Medicine Amsterdam UMC ‐ VU University Amsterdam Amsterdam the Netherlands; ^3^ Scientific Center for Transformation in Care and Welfare (Tranzo) University of Tilburg Tilburg the Netherlands

**Keywords:** comprehensive geriatric assessments, integrated care, older people, scoping review

## Abstract

In many integrated care programs, a comprehensive geriatric assessment (CGA) is conducted to identify older people's problems and care needs. Different ways for conducting a CGA are in place. However, it is still unclear which CGA instruments and procedures for conducting them are used in integrated care programs, and what distinguishes them from each other. Furthermore, it is yet unknown how and to what extent CGAs, as a component of integrated care programs, actually reflect the main principles of integrated care, being comprehensiveness, multidisciplinarity and person‐centredness. Therefore, the objectives of this study were to: (a) describe and compare different CGA instruments and procedures conducted within integrated care programs for older people living at home, and (b) describe how the principles of integrated care were applied in these CGAs. A scoping review of the scientific literature on CGAs in the context of integrated care was conducted for the period 2006–2018. Data were extracted on main characteristics of the identified CGA instruments and procedures, and on how principles of integrated care were applied in these CGAs. Twenty‐seven integrated care programs were included in this study, of which most were implemented in the Netherlands and the United States. Twenty‐one different CGAs were identified, of which the EASYcare instrument, RAI‐HC/RAI‐CHA and GRACE tool were used in multiple programs. The majority of CGAs seemed to reflect comprehensiveness, multidisciplinarity and person‐centredness, although the way and extent to which principles of integrated care were incorporated differed between the CGAs. This study highlights the high variability of CGA instruments and procedures used in integrated care programs. This overview of available CGAs and their characteristics may promote (inter‐)national exchange of CGAs, which could enable researchers and professionals in choosing from the wide range of existing CGAs, thereby preventing them from unnecessarily reinventing the wheel.

**What is known about this topic**

- Integrated care programs for older people living at home often include a comprehensive geriatric assessment (CGA) to increase the understanding of an older person's care needs and preferences.
- A complete overview of CGAs instruments and procedures used in integrated care is lacking. Also knowledge about how principles of integrated care are being applied in CGAs is limited.

**What this paper adds**

- This study demonstrates that there is little overlap in CGA instruments and procedures used in integrated care programs, and shows their similarities and differences.
- This overview supports exchange of knowledge on CGAs available in integrated care between researchers and professionals, which could enable them applying existing CGAs in their own contexts.

## 1 INTRODUCTION

Older people want to stay independent and live in their homes and communities until old age (Gillsjö, Schwartz‐Barcott, & von Post, 2011; Wiles, Leibing, Guberman, Reeve, & Allen, 2011). As the prevalence of multiple chronic conditions and disability increases with age, older people who live at home may suffer from limitations in the physical, cognitive, psychological, social and/or environmental domains of life (Hoogendijk et al., 2014; Lette et al., 2017). These limitations may challenge older people's social participation and independent living, and result in complex health and social care needs. There is growing evidence that integrated health and social care is a promising approach to address such care needs (Boult et al., 2009; De Bruin et al., 2012; Gress et al., 2009; Hopman et al., 2016; Mattke, Seid, & Ma, 2007; Wagner et al., 2005). Based on the Chronic Care Model and related models, we define integrated care as those initiatives that proactively seek to structure and coordinate care for older people in their own home environments, centred around their needs (Barr et al., 2003; Boult et al., 2009; De Bruin et al., 2012; Epping‐Jordan, Pruitt, Bengoa, & Wagner, 2004; Raleigh et al., 2014; Wagner et al., 2005).

Integrated care programs for older people living at home vary in types and numbers of intervention components. Most programs comprise frailty screening, multidisciplinary consultation meetings, case management, individualised care plans and follow‐up contacts to monitor the status of older people and the implementation of personalised care plans (Boult & Wieland, 2010; Eklund, Wilhelmson, Gustafsson, Landahl, & Dahlin‐Ivanoff, 2013; Hoogendijk, 2016; Looman, Fabbricotti, de Kuyper, & Huijsman, 2016). Another prevalent component within these programs to foster integration of care is the comprehensive geriatric assessment (CGA), also referred to as needs assessment, multidimensional assessment or geriatric assessment (Boult & Wieland, 2010; Eklund & Wilhelmson, 2009; Eklund et al., 2013; Hoogendijk, 2016; Kodner & Spreeuwenberg, 2002; Looman et al., 2016; Looman, Huijsman, & Fabbricotti, 2018; Oliver, Foot, & Humphries, 2014; Pilotto et al., 2017). The CGA can be defined as a multidimensional and interdisciplinary diagnostic process to identify older person's capacities, problems and needs, of which outcomes can serve as input for the development of a coordinated and shared plan for care and support and long‐term follow‐up (Rubenstein, Stuck, Siu, & Wieland, 1991). Through providing a uniform process for assessing needs, a CGA may promote shared understanding of older people's needs, common professional language and agreed‐upon practices and standards, which are considered essential ingredients in integrated care (Kodner & Spreeuwenberg, 2002). A CGA helps health and social care professionals to understand problems and care needs older people experience, so that services professionals provide are matched to the needs and preferences of the people they serve (Hoogendijk et al., 2014; Stijnen, Duimel‐Peeters, Jansen, & Vrijhoef, 2013). Also for older people themselves, it is important to understand and acknowledge their (unmet) care needs, as this is expected to increase their involvement in and control over their care and support. This in turn may support them to keep living in their own home environments (Chen & Thompson, 2010; Stijnen, Duimel‐Peeters, et al., 2013). Furthermore, a CGA enables proactive and early identification and management of care needs. Such a (preventive) assessment coupled with a plan for care and follow‐up may have significant benefits for older people's well‐being and independence including a reduction of hospital admissions, moves into long‐term care, falls and long‐term mortality (Beswick et al., 2008; Melis, Adang, et al., 2008; Stuck, Siu, Wieland, Adams, & Rubenstein, 1993).

In the wide range of integrated care programs for older people living at home, different CGA instruments and procedures (i.e. standardised processes that were adhered to when undertaking a CGA) for conducting a CGA are in place. Although some studies reflect the variety of CGAs in integrated care (Hoogendijk, 2016; Looman et al., 2018), a complete overview of CGAs instruments and procedures used in the context of integrated care for older people living at home is not yet available. Such an overview would provide insight into the characteristics of the various available CGA instruments and procedures for conducting them in integrated care programs, and allow for comparison between CGAs.

Additionally, for CGAs as an intervention component of an integrated care program, we hypothesise that the principles of integrated care are reflected in this specific component. However, it is yet unknown how and to what extent CGAs actually adhere to these principles. Such insights may help researchers and professionals with selecting a CGA from the range of existing instruments and procedures, and increase their awareness of differences between CGAs and their applicability in different integrated care contexts. We therefore conducted a review of the literature in order to: (a) describe and compare different CGA instruments that are being used in integrated care programs for older people living at home, and procedures for conducting them, and (b) describe how the principles of integrated care are being applied in these CGAs.

## 2 METHODS

In order to explore, map and synthesise information on CGAs in the context of integrated care, a scoping review was conducted. The methodological steps outlined in the Arksey and O'Malley framework for conducting scoping reviews were followed to undertake such a review (Arksey & O'Malley, 2005). This process included five steps: (i) identifying the research question, (ii) identifying relevant studies, (iii) selecting appropriate studies, (iv) charting the data and (v) collating, summarising and reporting the results (Arksey & O'Malley, 2005).

### 2.1 Identifying research questions

The research questions were as follows:

1. What are the characteristics of the integrated care programs in which CGAs are conducted?
2. Which CGA instruments and procedures are used in integrated care programs for older people living at home?
3. What are differences and similarities between CGA instruments and procedures to conduct a CGA?
4. How and to what extent are principles of integrated care reflected in CGA instruments and procedures for conducting a CGA?

### 2.2 Identifying relevant studies

A systematic literature search was conducted in the electronic databases Medline/PubMed, Embase and Scopus. The databases were searched for scientific English language papers published between January 2006 and June 2018 describing integrated care programs for older people living at home.

Table 1 shows the search terms that were used to search in the titles and/or abstracts of potentially relevant papers, in the databases Medline/PubMed, Embase and Scopus.

**Table 1:** Search terms used in the databases Medline/PubMed, Embase and Scopus

*care coordination OR case management OR comprehensive health care OR continuity of patient care OR critical pathways OR delivery of integrated health care OR disease management OR guided care OR integrated care OR long term care OR managed care, managed care programs OR patient care management OR patient care planning OR patient care team OR patient centred care OR patient oriented care OR shared care OR transmural care OR integrated health and social care OR integrated care pathways OR multidisciplinary program OR comprehensive care program OR proactive care OR interdisciplinary program OR person‐centred care OR chronic care model OR person‐centredness OR patient‐centredness*
AND
*needs OR needs assessment OR geriatric assessment OR risk prediction OR risk factors*
AND
*frailty OR multimorbidity OR multiple chronic conditions OR multiple needs OR complex needs OR frail OR vulnerable OR chronic disease OR chronic illness OR co‐morbidity OR geriatric care*
AND
*older people OR elderly people OR older adults OR elderly OR aged OR aging*
AND
*living in the community OR living at home OR community‐dwelling OR home dwelling OR independent living OR home care services OR home nursing*

In addition to the search in the electronic databases, relevant papers were identified through reference tracking.

### 2.3 Selecting appropriate studies

Two reviewers (AS and PvG) independently reviewed the papers yielded by the search for their relevance by screening their titles and abstracts. When considered relevant by both reviewers, the full‐text paper was retrieved and reviewed for its relevance again. Any disagreement between the reviewers was resolved by consulting a third reviewer (SdB) to reach consensus.

In this study, three key components were selected, which were considered to represent the main principles of integrated care (De Bruin et al., 2012; Hopman et al., 2016; Kodner & Spreeuwenberg, 2002): (a) comprehensiveness: focus on problems and (care) needs in different domains of life (e.g. physical, cognitive, psychological, social and environmental), (b) multidisciplinarity: involvement of health and social care professionals from multiple disciplines and sectors and (c) person‐centredness: active involvement of older people and their informal carers in decision‐making and planning their care process, and putting their capacities, needs and preferences at the centre.

For the present study, papers were eligible if they met the following inclusion criteria:

1. The integrated care program itself as described in the paper complied with our main principles of integrated care: (a) comprehensiveness, (b) multidisciplinarity and (c) person‐centredness;
2. The integrated care program aimed to address frailty or complex/multiple health and social care needs in multiple domains of life which may benefit from an integrated care approach;
3. The target population of the integrated care program included people aged 65 years and older living at home. Living at home means living in their own homes or in some form of assisted living. Also papers that focus on people enrolled in an integrated care program that was started during hospitalisation and was continued after people's discharge in their home environment were included;
4. The integrated care program contained a CGA and consisted of multiple components or interventions (e.g. CGA is followed by designing and executing an individualised care plan);
5. The paper concerned a study protocol of the integrated care program or an intervention study evaluating the impact of a program. These papers were expected to provide most extensive information about the CGA or provided a reference to a paper in which such information could be found. In case the CGA was described elsewhere, the paper which was referred to was also included in this study. A description included the CGA instrument and/or procedures for conducting the CGA. Procedures were the standardised processes that were adhered to when undertaking a CGA including established guidelines and experiences from practice.

Duplicate studies, papers not written in English or not retrievable and non‐scientific papers (e.g. an editorial) were excluded.

For this study, the methodological rigour of the included studies was not evaluated (Arksey & O'Malley, 2005). This was in line with the principles of scoping reviews, as the focus of our study was to map existing literature on a specific topic (in this case CGAs in integrated care) rather than to evaluate the impact of a particular intervention.

### 2.4 Charting the data

Two authors (AS and PvG) extracted relevant data from the studies included. A standard data extraction sheet was developed to capture all relevant aspects. The identified integrated care programs were reviewed as to their main characteristics: country, program objective, target group, setting and involved professionals. In accordance with our research aims and objectives, the detailed description of the CGA instruments and the procedures for conducting the CGA included name of CGA instruments/tools, its origin, composition, extensiveness, information available about the content of the CGA, format to conduct the CGA, validity, reliability, feasibility, location where the CGA was administered, duration and required staff training for conducting the CGA. The decision to extract this information was made after extensive review of included studies to determine most important features of CGAs and a subsequent discussion between authors. In addition, data about the way and the extent CGAs reflected the main principles of integrated care were extracted from the papers, being comprehensiveness, multidisciplinarity and person‐centredness.

### 2.5 Collating, summarising and reporting the results

The findings of this review are presented using a narrative style as well as using tables to systematically present all specific details about the CGA instruments and procedures, and the incorporation of the principles of integrated care in the CGAs. The results section will first describe the output of the literature search. Then, the characteristics of selected integrated care programs will be described to provide insight into the context in which CGAs were conducted. Then, the results will report characteristics of the CGA instruments and procedures for conducting them, their differences and similarities and the extent to which they incorporate principles of integrated care.

## 3 RESULTS

### 3.1 Study retrieval

**Figure 1:**
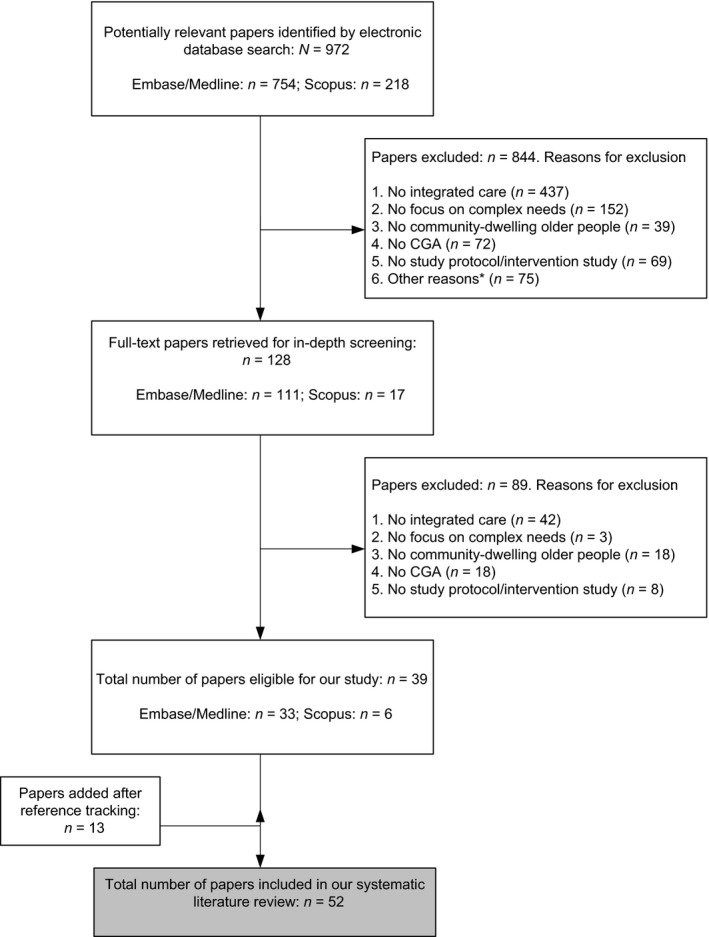
Flow diagram of literature screening process. *Reasons for exclusion other than the above, for example, duplicates, papers were not written in English, papers could not be retrieved or identified documents were non‐scientific papers

A total of 972 articles were identified in the original literature search (Figure 1). On the basis of their titles and abstracts, 128 publications were selected for full‐text screening, of which 39 were included in this study. Thirteen papers were added after reference tracking to provide a more thorough description of the integrated care program and the CGA when the papers resulting from the literature search did not provide sufficient information. This resulted in a total of 52 scientific papers describing 27 integrated care programs and 21 different CGA instruments. For one integrated care program, we present both the original and the updated program (Kono et al., 2009; Kono, Izumi, Yoshiyuki, Kanaya, & Rubenstein, 2016). As the programs were rather different, they were included as two separate programs.

### 3.2 Characteristics of integrated care programs

Many included integrated care programs were implemented in the Netherlands (*n* = 12). The integrated care programs were also implemented in the United States (*n* = 5), Canada (*n* = 4), Japan (*n* = 2), France (*n* = 1), Germany (*n* = 1), New Zealand (*n* = 1) and Sweden (*n* = 1) (Table 2). All but two programs (Moore et al., 2012; Tracy, Bell, Nickell, Charles, & Upshur, 2013) explicitly focused on improving outcomes in older people. Eleven programs additionally focused on: (a) improving quality of care, (b) improving efficiency of care and/or (c) enhancing skills of professionals and students. Except three programs, all included integrated care programs specifically targeted at older people being frail, including people with multiple health and social care needs and problems and people at (increased) risk of an adverse outcome. Three programs focused on either potentially frail older people (Stijnen, Duimel‐Peeters, et al., 2013), did not exclusively focus on frail older people (Spoorenberg et al., 2013; Spoorenberg, Wynia, Uittenbroek, Kremer, & Reijneveld, 2018; Uittenbroek, Kremer, Spoorenberg, Reijneveld, & Wynia, 2016) or addressed a frail population but did not explicitly mention frailty in the inclusion criteria of the study (Parsons, Sheridan, Rouse, Robinson, & Connolly, 2013).

**Table 2:** Characteristics of integrated care programs using CGA

Authors	Country	Integrated care program	Program objective	Setting and involved professionals	Target group
Blom et al., 2016; Hertogh, Deerenberg‐Kessler, & Ribbe, 1996, a	The Netherlands	ISCOPE (Integrated Systematic Care for Older People)	Restore, maintain or maximise functional independence, or to compensate for loss of autonomy by appropriate support.	Primary care setting. GP, PN Other professionals: HCN, PT, pharmacists, SW, specialists, nursing home physician, elderly care GP	Older people living in the community with a combination of somatic, functional, mental and social problems.
Boult et al., 2013; Boyd et al., 2008; Boyd et al., 2007, a	United States	GC (Guided Care)	Improve quality of life and promote the efficient use of resources.	Primary care PCP, RN Healthcare professionals working in emergency departments, hospitals, rehabilitation facilities, offices, nursing homes and at home Community resources: transportation services, Meals on Wheels, etc.	Older people with multimorbidity registered with involved primary care practice.
Bouman et al., 2008; Nicolaides‐Bouman, van Rossum, Kempen, & Knipschild, 2004, a	The Netherlands	Home visiting program	Maintain or improve the functional status of elderly and reduce the use of institutional care services.	Home care organisation HCN, PHN In‐home specialists within home care organisations: dietitian, diabetes specialist, OT; nurse geriatric specialist from local hospital GP Professional or community services	Older people living at home and with poor health.
Buurman et al., 2010; Buurman et al., 2016	The Netherlands	Transitional Care Bridge	Preserve physical functioning.	Transitional care: from hospital to primary care Geriatric consultation team: RN, geriatrician, clinical nurse specialist in geriatrics, PT, dietitian Hospital wards, other disciplines: pharmacist, OT GP, CN Other disciplines in primary care setting: dietitian, OT, elderly welfare consultant, PT, pharmacist Primary care geriatric consultancy team: GP, CN, consultant pharmacist, primary care PT, OT, elderly welfare consultant, social worker Geriatrician (in‐hospital consultant)	Older people acutely admitted to the department of internal medicine, hospitalised for at least 48 hr and at high risk of functional decline.
Counsell, Callahan, Buttar, Clark, & Frank, 2006; Counsell et al., 2007	United States	GRACE (Geriatric Resources for Assessment and Care of Elders)	Optimise health and functional status, decrease excess healthcare use and prevent long‐term nursing home placement.	Primary care setting Support team: NP, SW Interdisciplinary team: geriatrician, pharmacist, PT, mental health SW, community‐based services liaison PCP	Low‐income older people living in the community.
Daniels et al., 2011; Metzelthin et al., 2010; Metzelthin et al., 2013	The Netherlands	PoC (Prevention of Care)	Reduce disability and prevent (further) functional decline.	Primary care setting Core team: GP, PN (CM) Additional team members: OT, PT, other community care professionals or hospital professionals	Frail older people living in the community.
Faul et al., 2009	United States	GEMS (Geriatric Evaluation and Self‐Management Services)	Identify evidence‐based geriatric assessment and brief, prevention‐oriented intervention practices; teach these practices and related skills to interdisciplinary teams of SW and PT students and professionals; use these trained people to provide these interdisciplinary services to older people living in the community to complement the traditional care sought by patients; evaluate effectiveness of both these training and service components.	Interdisciplinary team: PT professional, PT student, SW student Other GEMS SW and PT students and professionals PCP Community resources: volunteer opportunities, support groups, etc.	Older people living in the community with one or more chronic illnesses.
Fleischer et al., 2008; Brettschneider et al., 2015, a	Germany	Preventive home visits (Brettschneider et al., 2015)	Reduce incidence of nursing home admissions in a cost‐effective way.	Nursing scientist, psychologist, sociologist Multi‐professional team (conference expert advisory group): nursing scientist, psychologist, geronto‐psychiatrist, nutritionist, social worker	Older people living at home or planned discharge to home and being impaired in at least three activities of daily living.
Hoogendijk et al., 2016; Muntinga et al., 2012	The Netherlands	GCM (Geriatric Care Model)	Improve quality of care, and subsequently improve their quality of life.	Primary care setting PCP, PN Multidisciplinary team: PN, PCP, pharmacist, geriatric team, other healthcare professionals: PT, etc. Geriatric team: geriatric nurse, elderly care physician Primary care professionals and representatives of various community‐based care organisations	Frail older people living in the community.
Van Hout et al., 2010	The Netherlands	Preventive home‐visiting program	Prevent functional decline, institutionalisation and mortality.	Primary care practices CN PCP Other visiting health professionals Local social and healthcare services	Frail older people living at home.
Kono et al., 2009	Japan	Preventive Home Visit program	Target‐specific care needs to provide efficient community‐based primary care.	Community‐based comprehensive care centres Home visitors: CN, CM, SW Community members, community care professionals, care management, community‐based service and urgent care: local government volunteers, PHN, social welfare financial services, etc. PCP, CM	Ambulatory frail older people living at home, certified as being in the two lowest levels of care need in the national insurance system.
Kono et al., 2016; Kono, Izumi, Kanaya, Tsumura, & Rubenstein, 2014, a	Japan	Updated Preventive Home Visit program	Improve or maintain quality of life and use appropriate long‐term care services.	Community‐based integrated care centres Home visitors: CN, CM, SW Long‐term, healthcare	Ambulatory frail older people living at home, certified as being in the two lowest care‐need levels in the national long‐term care insurance system.
Looman et al., 2014; Looman et al., 2016; Fabbricotti et al., 2013, a	The Netherlands	WICM (Walcheren Integrated Care Model)	Improve quality and efficacy of care given by their caregivers and health professionals.	Primary care setting GP, NP (CM) and secondary‐line geriatric NP (CM) Other health professionals: geriatric PT, geriatricians, pharmacists, district nurse, nursing home physician, mental health workers, SW, etc.	Frail older people living at home or in some form of assisted living.
Mazya et al., 2013	Sweden	AGe‐FIT (The Ambulatory Geriatric Assessment – a Frailty Intervention Trial)	Prevent hospital readmissions and functional deterioration in high‐risk older people.	Outpatient hospital care setting Interdisciplinary team: physicians, nurses, PT, OT, dietitian, SW, pharmacist Hospital departments, primary care centres	Older people living at home with multimorbidity and multiple hospital admissions in the previous year.
Melis, Van Eijken, et al., 2008; Melis et al., 2005, a; Richardson, 2001, a	The Netherlands	DGIP (Dutch Geriatric Intervention Programme)	Improving health‐related quality of life and promoting successful ageing.	Primary care setting PCP Intervention team: geriatric specialist nurse, geriatrician Other involved healthcare workers: HCN, PT, etc.	Older people living at home or in a retirement home with one or more limitations in cognition, (instrumental) activities of daily living or mental well‐being.
Moore et al., 2012	Canada	SCCP (Seniors Collaborative care Program)	Improve quality, efficiency, and coordination of care, and enhance geriatric and interprofessional skills for providers and learners.	Primary care setting Core team: FP, NP, registered PN Additional team members: pharmacist, dietitian, SW Visiting geriatrician Other FHC learners and practitioners Patient's main care team and community care providers	Frail older people living in the community at risk of falling and cognitive impairment.
Parsons et al., 2013; Parsons, Rouse, Robinson, Sheridan, & Connolly, 2012, a	New Zealand	Model of Restorative Home Care	Improve physical function and independence.	Home care Needs assessor from needs assessment agency RN (home care coordinator) Allied health professionals: OT, PT, speech‐language pathologist, dietitian	(Frail) Older people living in the community and being a new referral for home care.
Ploeg et al., 2010	Canada	Preventive primary care outreach intervention	Identify unrecognised problems and people at increased risk and link those people to appropriate health and social care.	Community primary care setting FP HCN Pharmacist, dietitian, PT Various community health and support organisations: home care services, meals on wheels, outpatient clinic, etc.	Older people at risk of functional decline and not receiving home care services.
Rogerson et al., 2006	United States	GRT (Geriatric Resource Team)	Maintain older people's independence and reduce institutionalisation within acute and long‐term facilities.	GRT team: nurse (CM), pharmacists, geriatricians, other professional disciplines PCP Community services	Disabled older people living at home.
Rosenberg, 2012	Canada	PIECH (Primary Integrated Interdisciplinary Elder Care at Home)	Improve quality of life, reduce caregiver burden, prevent and delay nursing home placement, improve and maintain functional status, prevent hospitalisation, facilitate home death, improve access to primary care, allow informed choices about intensity of medical interventions, teach medical students and residents care of the elderly in a home environment.	In‐home primary care setting Integrated team: PCP, nurse, PT Regional laboratory CN Home support services Family doctors from local clinics (on‐call PCPs) Hospitalists and medical specialists Community resources CM, CN and aides, and pharmacists	Frail older people living in the community.
Ruikes et al., 2012; Ruikes et al., 2016; Van Kempen et al., 2013, a	The Netherlands	CareWell	Prevent functional decline, improve quality of life and reduce or postpone hospital and nursing home admissions.	Primary care setting CN, research assistant Multidisciplinary team: GP, CN (CM), gerontological SW (CM), elderly care physician Pharmacist	Older people being (possibly) frail living in the community.
Schubert, Myers, Allen, & Counsell, 2016	United States	GRACE (Geriatric Resources for Assessment and Care of Elders) at VAMC (Veterans Affairs Medical Center)	Reduce acute care usage and lower costs at VAMC.	Primary care at VAMC Support team: NP, SW Interdisciplinary team: geriatrician, pharmacist, psychologist, mental health liaison PCP	High‐risk older veterans living at home or in assisted living and discharged home from an acute hospitalisation.
Spoorenberg et al., 2013; Spoorenberg et al., 2018; Uittenbroek et al., 2016; Spoorenberg, Reijneveld, et al., 2015, a	The Netherlands	Embrace	Support older people to age in place by providing person‐centred, integrated, proactive and preventive care and support.	Primary care setting Elderly care team: GP, elderly care physician, district nurse (CM), SW (CM) Professionals and volunteers	All older people living in the community (people are classified into three risk profiles: robust profile; frail profile; complex care needs profile)
De Stampa et al., 2014; Vedel et al., 2009; Morris et al., 1997, a	France	COPA (Coordination of care for the elderly)	Optimise patient care trajectories and in particular, decrease unplanned hospitalisations.	Primary healthcare setting Two‐person team: PCP, gerontology nurse (CM) Community‐based geriatrician Multidisciplinary primary care team: primary healthcare professionals Health and social services Psychologist Hospital team (hospitalist physicians)	Older people living in the community and being very frail with complex health and social needs.
Stijnen, Duimel‐Peeters, et al., 2013; Stijnen, Jansen, et al., 2013, a	The Netherlands	[G]OLD (Getting OLD the healthy way)	Improve health‐related quality of life and reduce disability.	Primary care setting GP, PN Primary healthcare and other care and/or well‐being organisations: PT, OT, etc.	“Apparently healthy” (potentially frail) older people living in the community.
Suijker et al., 2012; Suijker et al., 2016	The Netherlands	FIT (Functional decline In Transition)	Prevent functional decline in older people living in the community.	Primary healthcare setting GP, RN Other healthcare professionals: OT, PT, elderly welfare consultants, etc.	Older people living in the community at increased risk for functional decline.
Tracy et al., 2013	Canada	IMPACT (Interprofessional Model of Practice for Aging and Complex Treatments)	Design and evaluate a new interprofessional model of care and explore the potential of this new model as an interprofessional training opportunity.	Primary care setting Comprehensive team: FP, FP resident, CN, pharmacist, PT, OT, dietitian and a community SW	Older people living in the community with complex healthcare needs.

Abbreviations: CGA, Comprehensive Geriatric Assessment; CM, case manager; CN, community nurse; FP, family physician; GP, general practitioner; HCN, home care nurse; NP, nurse practitioner; OT, occupational therapist; PCP, primary care physician; PHN, public health nurse; PN, practice nurse; PT, physical therapist RN, registered nurse; SW, social worker.

a Added after reference tracking.

The settings in which the integrated care programs were implemented varied from the primary care setting to home care organisations, hospitals and specialised centres (e.g. Veterans Affairs medical centres, community‐based integrated care centres). Eighteen programs were implemented in primary care practices. In about half of the programs, all implemented in the primary care setting, the CGA process relied on a core team consisting of a general practitioner (GP; or other type of physician) and a nurse. The GP (or physician) was responsible for the care and treatment older people receive. The nurse was often designated as case manager and responsible for coordination and continuity of care across different settings and providers of care. In many programs, the core team was supported by a multidisciplinary team of social workers, elderly welfare consultants, physical therapists, occupational therapists, dietitians, pharmacists, (specialist) nurses, psychologists, geriatricians, nursing home physicians, psychiatrists and other medical specialists. The composition of a multidisciplinary team differed, ranging from a team of exclusively medical professionals to a team of an equal number of health and social care professionals. In most programs, the core and/or multidisciplinary team also consulted or referred to other professionals or community organisations providing services beyond the (professional) expertise of the core and/or multidisciplinary team, for instance medical specialists, social workers or meal delivery organisations.

### 3.3 Characteristics of CGAs (i.e. instruments and procedures) and differences and similarities between CGAs

#### 3.3.1 Instruments

Many different CGA instruments were used in the included integrated programs. In the 27 integrated care programs included in this study, 21 different CGA instruments were identified. All details with regard to the CGAs can be found in Table S1. Most programs developed or selected a unique CGA instrument, except for the programs using the RAI‐HC/RAI‐CHA, EASYcare instrument and GRACE tool. These instruments (i.e. EASYcare instrument, RAI‐HC/RAI‐CHA and GRACE tool) were used in different included programs (Table S1 – column ‘CGA instrument/tool’). The EASYcare instrument was used in five programs, all in the Netherlands. The RAI‐HC/RAI‐CHA was used in four programs, in the Netherlands, Canada and France. The GRACE tool was used in two US programs. The EASYcare instrument was either used as a standalone CGA instrument or as part of a composite CGA instrument consisting of different instruments including the EASYcare instrument.

The origin of the instrument differed across CGAs: (a) some instruments were developed to be used in different countries and healthcare settings, not specifically integrated care settings, such as the EASYcare instrument and the RAI‐HC/RAI‐CHA, (b) other instruments were based on or adapted from an existing assessment tool or format which had previously been developed for a different purpose or target group, such as the CGA FIT, GRT tool, SASPC system, [G]OLD instrument and Geriatric ICF Core Set and (c) for all other instruments, papers disclosed little or no details of the origin of the CGA instrument.

In most programs, the CGA encompassed a bundle of different types of instruments and questions to gain insight into the problems and care needs of the older person, for instance: (a) validated instruments, (b) questions derived from clinical guidelines or literature, (c) newly developed questionnaires or questions formulated by an expert panel or research team, (d) biomedical measurements and (e) items scoring physical home environment. CGAs were different in the degree of extensiveness. For instance, one CGA consisted of a 20‐page assessment covering more than 300 questions (Rogerson, Weiss, & Phillips, 2006), while another CGA seemed to be less extensive, including a standardised assessment for falls and cognition, and identification of goals and preferences (Moore et al., 2012).

Information provided about the exact content of the instrument varied between CGAs. For five CGA instruments, the content of the CGA was available in (the appendix of) the original study or in a study cited in the original study (Buurman et al., 2016; Buurman, Parlevliet, van Deelen, de Haan, & de Rooij, 2010; Fleischer et al., 2008; Ruikes et al., 2012, 2016; Stijnen, Duimel‐Peeters, et al., 2013; Suijker et al., 2012, 2016). For the other CGAs, the original study did not provide or only partially provided the exact content of the CGA nor referred to other papers describing this information.

#### 3.3.2 Procedures

Integrated care programs established procedures for conducting a CGA in order to enhance consistency in application. For a number of CGAs, papers explicitly mentioned that a structured format was followed for conducting the CGA, which means that instruments and questions were defined prior to the assessment and were preferably administered to each older person that was assessed (Table S1—column “Procedures”). Some CGAs encompassed a basic assessment that was conducted for each older person, which could be supplemented with additional assessments when considered necessary and desirable by the involved staff, older person and informal carer.

Three CGA instruments were tested for their validity and reliability (De Stampa et al., 2014; Ruikes et al., 2012, 2016; Van Hout et al., 2010; Vedel et al., 2009). For four CGAs, it was explicitly indicated that the instrument was tested for its feasibility (Daniels et al., 2011; Metzelthin, van Rossum, de Witte, Hendriks, & Kempen, 2010; Metzelthin et al., 2013; Ruikes et al., 2012, 2016; Stijnen, Duimel‐Peeters, et al., 2013; Suijker et al., 2012, 2016). For nine CGAs, the average duration of conducting the assessment was described, which ranged from 30 min to 3 hr. The location where a CGA was conducted differed between the integrated care programs. Most CGAs were conducted during a visit at the older people's home. In few programs, CGAs were conducted during hospital admission, via a telephone call, or during a visit at a clinic or centre. Information on whether or how (frequently) a CGA was used to monitor changes in functioning and care needs of older individuals was limited in the papers. For two programs, papers explicitly mentioned that the nurse involved used a digital instrument to conduct the CGA (Hoogendijk et al., 2016; Muntinga et al., 2012; Van Hout et al., 2010).

Particularly professionals from Dutch integrated care programs attended a training program or (refresher) course on conducting a CGA and related topics, including care planning, goal setting, stimulating self‐management, motivational interviewing, case management, interdisciplinary collaboration and the use of a computer for the assessments. Training was usually provided to the nurse responsible for conducting the CGA and acting as case manager in the program, although in several programs also other professionals, such as the GP or entire multidisciplinary team, attended a training program.

### 3.4 Adherence to principles of integrated care

#### 3.4.1 Comprehensiveness: life domains addressed in CGA

As described in Table S1 (column “Comprehensiveness”), all CGAs covered problems and care needs in different domains life (e.g. physical, psychological, functional, social and environmental domain), and can as such, based on our operational definition, be considered as comprehensive. CGAs varied in the number and type of life domains they addressed. Most frequently covered domains in CGAs were related to physical, functional, psychological, cognitive and social functioning, as well as medication. Examples of domains that were less frequently covered were abuse signs (e.g. physical or emotional abuse; Kono et al., 2009; Van Hout et al., 2010), finances (Fleischer et al., 2008; Suijker et al., 2012, 2016), and providing informal care (Stijnen, Duimel‐Peeters, et al., 2013). In addition to addressing different life domains, some CGAs included a section in which concerns, priorities and goals of the older person and the informal carer were explicitly assessed.

The different domains that were addressed during the assessment were described for all CGAs except six. Some papers described the domains and its subdomains in detail (e.g. psychological domain covers cognition, delirium, depression, anxiety and dependency), whereas most papers only mentioned the elements covered by the CGA without further distinguishing of domains and subdomains. Furthermore, domains were defined in different ways in CGAs. For example, in some CGAs, cognitive functioning was part of the psychological/mental domain (Kono et al., 2016; Stijnen, Duimel‐Peeters, et al., 2013; Suijker et al., 2012, 2016), while in other CGAs, cognition was a domain separate from the psychological/mental domain (Boult et al., 2013; Boyd et al., 2008; Ruikes et al., 2012, 2016).

#### 3.4.2 Multidisciplinarity: professionals involved in CGA

CGAs differed in the extent to which they seemed to work in a multidisciplinary way during the inventory of the older person's situation and the development of the care plan. Depending on the integrated care program, the roles of professionals from different organisations and disciplines in conducting the CGA can be roughly divided into three different ways (Table S1—column “Multidisciplinarity”): (a) a nurse (practitioner) conducted the CGA, in some cases in collaboration with or under supervision of a GP, higher qualified nurse or social worker. In some of these programs, additional assessments by the nurse, GP or professionals from other disciplines were conducted when considered necessary, (b) all members of the multidisciplinary team conducted a part of the CGA which was related to their own professional expertise or (c) an assessor designated for performing the assessment conducted the CGA (but was not further involved in developing the care plan or providing care and support for the older person).

In line with the objective of a CGA to serve as input for developing a care plan, in each initiative, an individual care plan or recommendations for care and support were developed. They included identified problems, care needs, goals and actions to be taken, and were based on the outcomes of the assessment. The steps that were taken to develop a care plan differed between integrated care programs: (a) a nurse or GP formulated the care plan, (b) a nurse presented a draft care plan to the GP or multidisciplinary team to discuss outcomes of the CGA and the initial care plan before finalising it, (c) all core or multidisciplinary team members met to discuss the outcomes of the CGA and together formulated a care plan and decided on further action to be taken, or (d) professionals who were involved in the development of a care plan were not explicitly mentioned in the paper.

To sum up, healthcare professionals (GP, nurses, allied healthcare professionals) were always involved in the assessment of care needs and preferences and the development of a personal care plan. Social care professionals (social services, community organisations) were less frequently involved in these stages.

#### 3.4.3 Person‐centredness: involvement of older people and their informal carers in CGA

Across the programs, older people and their informal carers were involved in different ways in conducting the CGA and developing the care plan (Table S1—column “Person‐centredness”). CGAs generally aimed to take needs and preferences of the older person into account in the processes of conducting a CGA and developing the care plan, although the manner of working in a person‐centred way varied between CGAs: (a) in the majority of the programs, older people were encouraged to share their concerns, priorities and goals they would like to achieve. As such, professionals were supposed to develop care plans or recommendations in a way consistent with older people's needs and preferences, and (b) in a few programs, professionals developed care plans or recommendations based on the outcomes of the CGA without explicitly involving older people and their informal carers in conducting the CGA and developing the care plan.

After formulating the individually tailored care plan, in almost all programs, the professionals discussed the specifics of the care plan with older people and collaborated with them to review and modify it. In two of these programs, the care plan was only implemented after final approval by the older person (Looman, Fabbricotti, & Huijsman, 2014; Looman et al., 2016; Spoorenberg et al., 2013, 2018; Uittenbroek et al., 2016). In seven programs, older people were responsible for implementing activities and recommendations that were established in the care plan (Boult et al., 2013; Bouman, Van Rossum, Ambergen, Kempen, & Knipschild, 2008; Boyd et al., 2008; Faul et al., 2009; Fleischer et al., 2008; Ploeg et al., 2010; Rogerson et al., 2006; Suijker et al., 2012, 2016).

To encourage active involvement of the older person and informal carer in the decision‐making and planning of their care process, some papers explicitly indicated “strategies” to stimulate professionals to do so during conducting a CGA: building trust with older people and their informal carers (for instance by making an introduction visit), allowing older people time to talk and using motivational interviewing techniques (for instance summarising and validating given answers). Also developing a user‐friendly care plan could be part of this. In addition, some programs aimed to empower older people and their informal carers by providing self‐management support including coaching, advice, education and health promotion materials.

Altogether, the majority of CGAs seemed to address comprehensiveness, multidisciplinarity and person‐centredness, although not all CGAs reflected all three principles of integrated care to the same extent. Some CGAs reflected one of the principles less extensively than other CGAs. The CGAs conducted in the integrated care programs Embrace (Spoorenberg et al., 2013, 2018; Uittenbroek et al., 2016) and IMPACT (Tracy et al., 2013) seemed to incorporate the three principles most extensively, and as such showed how (extensively) the integrated care principles may be reflected in CGAs. These CGAs aimed to cover a broad range of health and social care needs and problems in different domains of life, involve professionals from multiple disciplines and sectors—including social workers—during the CGA and the development of the care plan, and actively include the older person and informal carers in the decision‐making and planning their care process.

## 4 DISCUSSION

The aim of this study was to describe and compare CGA instruments that were used in integrated care programs for older people living at home, and procedures for conducting them. There is a vast amount of literature describing CGAs conducted in integrated care programs. Some of these studies address the heterogeneity of CGAs used in integrated care (Hoogendijk, 2016; Looman et al., 2018). However, a systematic overview of CGA instruments and procedures and of their differences and similarities was lacking. This scoping review addressed these gaps (Foreman, Thomas, & Gardner, 2004; Hermans et al., 2014), and is unique in focusing on older people living at home in the context of integrated care. In addition, it was unknown how and to what extent CGAs, being a crucial intervention component of many integrated care programs (Boult & Wieland, 2010; Hoogendijk, 2016; Kodner & Spreeuwenberg, 2002), reflected the principles of integrated care. We therefore also aimed to describe how principles of integrated care were applied in existing CGAs.

### 4.1 Instruments and procedures

In total, our review yielded 27 integrated care programs and 21 different CGA instruments. Twelve of 27 integrated care programs were implemented in the Netherlands. This relatively large number can be explained by the Dutch National Care for the Elderly Programme which took place between 2008 and 2016. This Dutch program aimed to improve care and support for older people through funding of several research and implementation projects focused on developing a more proactive and integrated health and social care system for frail older people (Hoogendijk, 2016; Lutomski et al., 2013). Nine of these projects, with eight different CGA instruments and procedures, were included in this study (Blom et al., 2016; Buurman et al., 2016, 2010; Daniels et al., 2011; Hoogendijk et al., 2016; Looman et al., 2014, 2016; Metzelthin et al., 2010, 2013; Muntinga et al., 2012; Ruikes et al., 2012, 2016; Spoorenberg et al., 2013, 2018; Stijnen, Duimel‐Peeters, et al., 2013; Suijker et al., 2012, 2016; Uittenbroek et al., 2016).

As shown in this study, many different CGA instruments were used to identify older people's problems, care needs and preferences. Most programs developed or selected a unique CGA instrument, except for the programs using the RAI‐HC/RAI‐CHA, EASYcare instrument and GRACE tool, as they were used in different programs included in this study. The reason for the variability of CGA instruments and procedures for conducting them in integrated care programs is largely unknown; arguments for developing new CGAs were often not presented. One possible explanation may be related to the limited knowledge about how older people's problems and care needs should best be assessed using a CGA. Existing CGA instruments and procedures may not satisfy requirements set by other researchers and professionals of the integrated care programs. As such, these researchers and professionals may develop alternative instruments which they, for instance, perceive to be more suitable for their specific context, or which explicitly involve the target population in the development of the instrument (Spoorenberg, Reijneveld, et al., 2015; Stijnen, Jansen, Vrijhoef, & Duimel‐Peeters, 2013). Moreover, the existing controversy over an agreed definition of frailty as well as the insufficient evidence on appropriate frailty screening, diagnostic instruments and effective interventions may explain the variety of CGAs (Rodríguez‐Laso et al., 2018). Due to ambiguities on how frailty can best be screened, assessed and managed, programs may have different ideas of how to identify and address problems and care needs, leading to the development of new CGA instruments and procedures that are in line with their understanding of these concepts. Another explanation for the amount of CGAs may be a lack of awareness among researchers and professionals of the existing range of CGA instruments and procedures for conducting them, causing them to develop new CGAs themselves. This overview of available CGAs and their characteristics may support exchange of CGA instruments and procedures between researchers and professionals, which may reduce efforts to develop new ones.

In addition to the variability of CGAs, there were differences in the amount of details provided about the CGA instruments and procedures for conducting them across the included studies. Some papers for instance provided limited information about the specific domains that were addressed in the CGA or how exactly older people were involved during the process of conducting the CGA and developing and implementing the care plan. This hampered our goal of thoroughly describing and comparing different CGA instruments and procedures for conducting them, including their incorporation of integrated care principles. To enhance comparability, we recommend describing CGA instruments and procedures and their adherence to integrated care principles in more detail to obtain a more extensive overview.

### 4.2 Principles of integrated care

Our results show that all CGAs in this study may be considered comprehensive, which means that, to a greater or lesser extent, the importance of addressing older people's needs across different domains of life seems to be recognised (Lette et al., 2017). Care and support tended to primarily focus on physical health problems and needs (Kuluski, Ho, Hans, & Nelson, 2017; Lette, Baan, Van Den Berg, & De Bruin, 2015). However, gradually a broader concept of health including for instance also psychological, social and environmental life domains is being adopted in policy making, research, education and practice. The CGAs identified in this study are reflecting this broader health concept as well.

As for the principle of multidisciplinarity, multidisciplinary teams and the CGA concept of multidisciplinary assessment and management may have different potential benefits for the older person (Johansson, Eklund, & Gosman‐Hedström, 2010). Although social workers were involved in several CGAs, healthcare professionals were predominating the process of conducting the CGA and developing the care plan in this study. Social care professionals (social services, community organisations) have the ability to bring to the multidisciplinary team a unique perspective concerning the people to whom they provide care and support considering their specific expertise (Ambrose‐Miller & Ashcroft, 2016). Therefore, their involvement into multidisciplinary team working from the moment of identifying problems and care needs until addressing them with appropriate care and support is recommended.

With regard to the principle of person‐centredness, there is broad consensus that older people should be encouraged to participate actively in the process of care and decision‐making (Barry & Edgman‐Levitan, 2012). In most programs, older people and their informal carers were actively involved in conducting the CGA and developing the care plan. However, preferences for the degree and the way of participation in decision‐making may differ between older people (Belcher, Fried, Agostini, & Tinetti, 2006; Levinson, Kao, Kuby, & Thisted, 2005). Therefore, to actually work in a person‐centred way, older people should be offered the opportunity to be engaged in the process of their care, in which their preferences regarding the role they wish to play in their care are paramount (Levinson et al., 2005).

### 4.3 Methodological considerations

In this study, comprehensiveness, multidisciplinarity and person‐centredness were selected to represent the main principles of integrated care. We realise, however, that integrated care includes more than these three components. Still, these selected components are considered common aspects of integrated care and are therefore most likely to be explicitly addressed in papers describing integrated care programs (Hopman et al., 2016; Kodner & Spreeuwenberg, 2002). The authors felt that the application of stricter criteria would mean that highly relevant papers would not appear in our selection.

We performed a broad literature search to collect as many relevant papers as possible. One limitation of our search strategy is that we included scientific papers published from 2006 onwards. January 2006 was used as a starting point as from that moment onwards, literature on integrated care programs for older people living at home, in which a CGA may be conducted, became increasingly prevalent (De Bruin et al., 2012; Hopman et al., 2016). As such, we expected to find most relevant papers within the given timeframe. We only included scientific papers written in the English language because we would like the information described in the papers to be understandable for all readers. In addition, grey literature was not included in this study. Grey literature was generally not indexed in the electronic databases we used and was therefore excluded from the search (e.g. letters, notes, conference abstracts). Therefore, we may have missed relevant integrated care programs and their CGA instruments and procedures that were described before 2006, in other languages or in grey literature. Moreover, we did not include the entire range of identified CGAs that were used in integrated care programs for older people living at home. Several papers did not provide sufficient information about the CGA instruments and procedures used in the program, which made them ineligible for inclusion in this study.

To perform this study, we conducted a scoping review instead of a systematic review. Compared to systematic reviews which address more precise questions, scoping reviews also require transparent and rigorous methods in their conduct and are considered specifically helpful for scoping a body of literature on a certain topic (Munn et al., 2018). Since this study aims to map existing literature on CGAs in integrated care, conducting a scoping review seemed to be an appropriate choice. There are also certain limitations of conducting a scoping review that should be mentioned. We provided a comprehensive overview of available CGAs in integrated care. However, our scoping review did not provide empirical evidence for the effectiveness or cost‐effectiveness of CGAs in integrated care. Although it would be relevant for policy aims to increase our understanding of what works in what context, it will be challenging to establish a clear relationship between one specific component of integrated care programs and outcomes that may realistically be expected in the context of care for older people.

### 4.4 Recommendations for research and practice

This study provides an overview of available CGA instruments and procedures for conducting them, and insight into the extent to which they reflect principles of integrated care. Unfortunately, reasoning for the choice to select to develop a specific instrument mostly remains unclear. Still the overview in this study is useful in supporting the exchange of knowledge about CGAs between researchers and professionals, and helps them select a CGA appropriate for their context from the wide range of possible instruments and procedures. As such, this overview will enable researchers and professionals to make use of available CGAs where possible, in order to prevent them from unnecessarily reinventing the wheel. However, considering our methodological considerations, the current overview of available CGAs is not yet complete. More thorough descriptions of CGAs, as well as experiences with using them, are recommended to complete the overview of available CGAs in integrated care.

Furthermore, knowledge about which qualifications a CGA should preferably meet is still limited. While this study showed the extent to which CGAs reflect principles of integrated care, there are also other key features which could determine the choice for a CGA. These include, for example, the feasibility, validity and reliability of the CGA, whether users of CGAs have open access to the instrument and procedures or how easy it is to share CGA outcomes digitally between professionals. Also the extent to which CGAs is appropriate for monitoring changes in older people's functioning or living situation in relation to their care needs could be an important aspect. Further research is recommended to gain more insight into the value of such additional qualifications of CGAs in the context of integrated care for older people, and whether the existing range of CGAs meet these qualifications. These insights could then guide the adaption of existing instruments or, if necessary, the design of new ones.

Additionally, little is known about the experiences and views of older people and their informal carers with specific CGA instruments and procedures. These are, however, important when assessing existing CGAs (Spoorenberg, Reijneveld, et al., 2015). Therefore, we would also recommend to obtain more insight into what is important for older people with regard to the content and procedures of a CGA, taking into account the wide variation in preferences of individuals. Questions that could benefit from more research include, for example, whether older people consider a CGA relevant, and if so, what important themes are that should be addressed during a CGA (Kodner & Kyriacou, 2000; Lette et al., 2017), how long a CGA may last, who should be targeted (Stijnen et al., 2014) and which professional would be most suitable to build rapport between the older person and professionals (Spoorenberg, Wynia, et al., 2015; Van Kempen et al., 2012)?

## 5 CONCLUSION

This study shows that many different CGA instruments and procedures for conducting them are in place, each with a different perspective. CGAs vary in the way and the extent to which they reflect principles of integrated care. Most CGAs seemed to address comprehensiveness, multidisciplinarity and person‐centredness. However, in some cases, healthcare professionals were predominant or older people and their informal carers were not explicitly involved in the process of conducting the CGA and developing the care plan. This overview of characteristics of available CGAs supports knowledge exchange between researchers and professionals, although more thorough descriptions of CGAs and experiences with using them are necessary to further complete the overview. Sharing knowledge on CGAs available in integrated care could enable researchers and professionals apply existing CGAs in their own contexts.

## CONFLICT OF INTEREST

No conflicts of interest have been declared.

## AUTHOR CONTRIBUTIONS

All authors contributed to the study concept and design. AS and PvG carried out study selection. SdB was consulted in case of any disagreement during study selection. AS and PvG extracted relevant data from the studies included and analysed the data. AS drafted the manuscript and SdB, ML, CB, GN and PvG critically revised the manuscript. All authors read and approved the final version.
