# Supplementary material for: Comprehensive geriatric assessments in integrated care programs for older people living at home: A scoping review
Source: Health Soc Care Community. 2019 Jun 21;27(5):e549–66. doi: 10.1111/hsc.12793 (PMC6852049; doi:10.1111/hsc.12793)
Supplement: Supplementary file 1 [file HSC-27-e549-s001.docx]

**Supporting Material Table 1 (Table S1). Overview of integrated care programs including a CGA, CGA instruments procedures for conducting CGAs and incorporation of principles of integrated care in CGAs**

| **Authors** | **Integrated care program** | **CGA instrument/ tool** | **Procedures for conducting the CGA** | **Comprehensiveness (domains/ elements addressed in the CGA)** | **Multidisciplinarity (involvement of professionals in the CGA)** | **Person-centredness (involvement of older people and their caregivers in the CGA)** |
| --- | --- | --- | --- | --- | --- | --- |
| Blom, 2016 (Blom et al., 2016); Hertogh, 1996 (Hertogh, Deerenberg-Kessler, & Ribbe, 1996)* | ISCOPE | SASPC system   - Classification of problems in five categories, stemming from Dutch rehabilitation medicine | - GP and PN attend training on proactive integrated care, including designing, conducting and adjusting a care plan. | - Somatic domain - Psychological domain - ADL domain - Social domain - Communicative domain | - GP and/or PN (under supervision of GP) conducts CGA, defines care plan and formulates actions to be taken and evaluation plans for follow-up. - GP or PN involves other care professionals, for instance in multidisciplinary consultations, where needed. | - GP and/or PN explore wishes and expectations of the older person about goals to be achieved with older person and their informal carer(s). - The GP and/or PN, together with the older person, formulate actions to be taken and evaluation plans for follow-up. |
| Boult, 2013 (Boult et al., 2013); Boyd, 2008 (Boyd et al., 2008); Boyd, 2007 (Boyd et al., 2007)* | GC | - CGA includes bundle of instruments: inventories for impairment of IADL and ADL, questionnaires (e.g. Mini-Mental State Exam), screening questions (hearing impairment, falls, and urinary incontinence) and questions identifying highest priorities for optimizing health and quality of life. | - Nurse performs CGA during home visit. - Nurse completes an educational program that emphasizes skill development through interactive role-playing, supplemented by readings and brief lectures on comprehensive assessment and planning, coaching etc. | - Medical status - Functional status - Cognitive status - Affective status - Psychosocial status - Nutritional status - Environmental status | - RN conducts CGA and together with PCP develops care plan and provides involved health care professionals with a summary of the older person’s status and plans. - RN monitors older person by regular calls to detect and address problems and when problems appear, RN discusses them with PCP and takes action; RN reinforce adherence to care plan during monitoring calls - RN coordinates health care across continuum of care, including keeping PCP informed, and facilitating access to community resources. | - RN asks older person to identify highest priorities for optimizing health and quality of life during CGA. - RN and PCP align care plan with unique circumstances of older person, discusses this with older person and informal carer and modifies plan with their preferences, priorities and intentions. - Older person has a plan in lay language at their home. - RN is directly accessible by telephone to the older person and informal carer for questions and concerns. - RN promotes self-management, monitors the older person regularly and coaches the older person to practice healthy behaviors using motivational interviewing. |
| Bouman, 2008 (Bouman, Van Rossum, Ambergen, Kempen, & Knipschild, 2008); Nicolaides-Bouman, 2004 (Nicolaides-Bouman, van Rossum, Kempen, & Knipschild, 2004)* | Home visiting program | - CGA includes bundle of instruments: recording of problems and needs indicated by older person themselves, EASYcare instrument and additional checklists (e.g. vision, hearing and use of medication) to detect further problems including instruments for further diagnostic assessments (the get-up-and-go test). | - HCN conducts CGA during home visits, which takes between 60 and 90 minutes. - HCN and public health nurses are well trained to conduct home visits; HCN receives relevant training in communication skills and using assessment tools and took courses on several subjects, e.g., relevant geriatric health topics, behaviour change etc. | The paper(s) did not describe the domains that were addressed during the CGA. | - HCN conducts CGA, being supervised by PHN, and develops care plan including advice or information, and referral to professional or community services. - HCN can consult in-home specialists or nurse geriatric specialist, or refer to GP if necessary. - HCN sends overview of all treated problems to GP and invites to provide comments and suggestions. | - HCN starts CGA with recording problems and needs as experienced by older person. - HCN discusses activities of care plan in agreement with older person. - HCN provides advice or information about nutrition, social and physical activities, and home aids. - Older person is responsible to carry out planned activities supported by HCN. |
| Buurman, 2010 (Buurman, Parlevliet, van Deelen, de Haan, & de Rooij, 2010); Buurman, 2016 (Buurman et al., 2016) | Transitional Care Bridge | - Exact content of CGA is available in paper - In total, 21 components - CGA includes bundle of questions (e.g. Do you experience dizziness?) and instruments (e.g. Geriatric Depression Scale) - CGA starts CGA with screening on delirium, malnutrition, ADL functions, mobility and fall risk. | - RN conducts CGA at hospital admission and takes 30 minutes. - In cognitive impaired older people, part of the CGA is conducted by interviewing informal carer. - More intensive screening is conducted when considered necessary. - RNs who conduct the program receive additional training on geriatric care in the community, the transition from hospital to home, and provide patient-centered care and empowering the older person including motivational interviewing, before the start of the intervention. | - Somatic (e.g. mobility and stability) - Psychological (e.g. delirium) - Functional (e.g. ADL functioning) - Social (e.g. loneliness) | - RN conducts CGA and geriatric consultation team conducts more intensive screening. - RN discusses outcomes of CGA with geriatrician and clinical nurse specialist, and geriatric consultation team develops care and treatment plan, implements care in accordance with medical and nursing care at the ward, and consults other disciplines, such as OT or PT, when needed. - Team makes handover of care and treatment plan, and clinical nurse specialist coordinates handover to CN who visits older person before discharge. - CN discusses care plan with GP and enables additional support from other disciplines in primary care. - CN visits older person at home to discuss medication and care arranged during hospital admission, to reassess and adapt plan when necessary, and to discuss medication regimen from the hospital. - CN coordinates already implemented and new interventions, maintains contact with other disciplines in primary care and identifies new care needs in consultation with GP. - CN evaluates plan, the impact and results and discuss this with primary care geriatric consultancy team. - CN or GP consults in-hospital consultant when necessary. - GP has final responsibility for medical care. | - Nurse asks older person and informal carer to prioritize problems and to indicate most important goals to be achieved during and after hospital admission, after conducting CGA as input for discussion. - Geriatric consultation team discusses care plan (problems and treatment options) with older person and informal carer to enable them to make a well-informed decision about care and treatment plan - CN enables additional support at home depends on needs of older person and informal carer. - CN promotes the empowerment of older person and informal carer by providing psycho-education and ancillary services. |
| Counsell 2006 (Counsell, Callahan, Buttar, Clark, & Frank, 2006); Counsell, 2007 (Counsell et al., 2007) | GRACE | GRACE tool   - CGA includes a medical and psychosocial history, medication review, functional assessment, review of social supports and advance directives, and home safety evaluation. | - GRACE support team conducts CGA during home visit. - GRACE team completes special training in implementing the GRACE protocols and working as an interdisciplinary team during regular small group seminars. | The paper(s) did not describe the domains that were addressed during the CGA. | - Support team conducts CGA and present outcomes to interdisciplinary team to develop care plan. - Support team collaborates with PCP to review, modify and gain PCP approval of care plan. - Support team implements care plan collaborating with PCP and supported by interdisciplinary team. - Support team is responsible for care management and coordination and continuity of care among all health care professionals and sites of care. - Interdisciplinary team monitors care and support initiated. | - Support team encourages goal setting and self-care, teaches problem-solving skills and provides education using low-health-literacy materials; prepare older person to address problems and team suggestions during office visits. - Support team implements support plan consistent with the older person’s goals through contacts with older person and informal carers. - Older people receive an annual reassessment and follow-up visit to review the care plan; additional contacts occur as appropriate to implement the care plan; one phone contact per month and home visits after emergency visit or hospitalization. |
| Daniels, 2011 (Daniels et al., 2011); Metzelthin, 2010 (Metzelthin, van Rossum, de Witte, Hendriks, & Kempen, 2010); Metzelthin, 2013 (Metzelthin et al., 2013) | PoC | - CGA has a structured assessment format based on various tools derived from instruments proven their use in previous research (e.g. the EASYcare instrument). - Assessment was tested for its feasibility. | - PN conducts CGA during home visit which takes about one hour followed by half an hour of administration. - Additional assessments by other professionals are conducted when considered necessary. - Health professionals (PNs, GPs, PTs and OTs) receive relevant training sessions with regard to the aspects and basic principles of the intervention protocol, i.e. the screening procedure, self-management principles, client centeredness, motivational interviewing, interdisciplinary collaboration, assessment tools, parts of the toolbox, and referrals. | - Concerns and wishes expressed by the older person and informal carer. - Risk factors for disability (polypharmacy, mobility problems, falls, lack of physical activity, cognitive impairments, or mood problems). - Problems or concerns experienced in performing daily activities meaningful to the older person, such as gardening, visiting family/friends, etc. - Readiness to change. | - PN conducts CGA and discusses with GP if assessments by GP, PT OT or other specialized professionals are necessary. - Core team develops action plan or organizes a meeting with OT, PT and other disciplines to formulate plan. - Interdisciplinary team, inpatient and outpatient specialists deliver interventions, including referral to other disciplines. - PN as CM and older person (and informal carer) evaluate progress and agree on follow-up. | - PN uses motivational interviewing and validates answers during CGA. - Team takes into account meaningful activities, problems or concerns expressed by older person to formulate plan and can invite older person and informal carer to meeting. - After preliminary plan is developed, PN, older person and informal carer set an agenda and a list of goals and actions tailored to the specific needs and wishes of the older person and informal carer, using motivational interviewing - PN evaluates with older person and informal carer progress and need for support in following period. |
| Faul, 2009 (Faul et al., 2009) | GEMS | - CGA includes bundle of instruments: measures of basic demographic and family characteristics (General Assessment Questionnaire), screening measures (e.g. Mini-Cog Screening Tool), and home environment evaluation. | - PT student and SW student conduct CGA during two home visits which take about three hours - Due to professional requirements, PT professional accompany students during CGA. - SW reads the questions and the corresponding response options to the older person unless they prefer to complete the forms by themselves. The survey is in large font size to accommodate such a request. | - Basic demographic and family characteristics - Cognitive dysfunctions - Home environment (i.e. fall risk) - PT Patient Management Systems Review | - Interdisciplinary team conducts CGA, meets with other GEMS students and professionals to discuss outcomes of CGA and develop care plan and provides additional self-management support. - Interdisciplinary team sends outcomes of CGA and care plan to PCP and encourages older people to follow-up with PCP. | - Interdisciplinary team develops care plan based on the unique health status, as well as family and environmental factors of the older person. - Interdisciplinary team meets with older person to discuss outcomes of CGA, to discuss care plan and exercise plan, to demonstrate the exercise plan, and support participant in finalizing self-management care plan including community resources that could be used to maintain or improve health and mental health status. - Older person revises care plan in collaboration with interdisciplinary team to ensure individual needs and preferences are met. |
| Fleischer, 2008 (Fleischer et al., 2008); Brettschneider, 2015 (Brettschneider et al., 2015)* | Preventive home visits | - Exact content of CGA is available in paper - CGA includes bundle of existing instruments (e.g. Mini Nutrition Assessment) | - Nursing scientist, psychologist or sociologist conducts CGA during first home visit. | - Nutrition status - Impairment of sight and hearing - Urinary and bowel incontinence - Loss of functional muscle mass - Social activities - Housing conditions - Economical conditions - Polypharmacy - Cognitive abilities | - Nursing scientist, psychologist or sociologist conducts CGA - Conference expert advisory group works out recommendations for older person. - Nursing scientist, psychologists or sociologists conducts two follow-up visits for addressing the identified problems and presenting the recommendations, and boosting and evaluating implementation of recommendations. | - Conference expert advisory group develops individualized recommendations. - Nursing scientist, psychologist or sociologist visits older people for consultation and instruction of recommendations, and evaluation of the implementation of recommendations by older person. |
| Hoogendijk, 2016 (Hoogendijk et al., 2016); Muntinga, 2012 (Muntinga et al., 2012) | Geriatric Care Model | RAI-CHA | - PN conducts CGA during home visit. - PN receives training on motivational interviewing and clinical education on geriatric topics. - PN and members of geriatric team participate in one-day RAI workshop and refresher course RAI. - CGA is conducted using web-based version of RAI-CHA. | The paper(s) did not describe the domains that were addressed during the CGA. | - PN conducts CGA and review outcomes of CGA with PCP, develops care plan, and evaluates outcomes of actions listed in final care plan. - Geriatric team invites multidisciplinary team and other health care professionals where relevant to multidisciplinary consultation for complex cases. - Geriatric team organizes network meetings between primary care professionals and representatives of various community-based care organizations. | - PN explores the older person’s wishes regarding further management or care, informs about care options and stimulates active involvement in the decision making process; PN and older person formulate care goals and actions for the final care plan. - PN and older person evaluate outcomes of actions listed in care plan; regular contact by telephone, and if necessary, an additional home visit. - The older person’s own care wishes remain at the center of the decision making process. |
| Van Hout, 2010 (Van Hout et al., 2010) | Preventive home-visiting program | RAI-HC   - In total, 30 components - RAI-HC is validated multidimensional geriatric assessment instrument. - Care needs are assessed using a computerized instrument. | - CN conducts CGA during home visit and takes between 45 and 75 minutes. - CN enters assessment directly on laptop. - CN receives training during 2-day session about (computerized) CGA and care planning, local social and health care services, using a computer, consultation meetings, quality of care, etc. | - Preventive health - Pain - Medication management - Safety environment - Falls - Health promotion - Skin and food problems - Urinal incontinence catheter - Visual functioning - Communication - Social functioning - Nutrition - IADL/more formal care - Depression and anxiety - Cognition - Oral/dental health - Fecal incontinence - ADL revalidation potential - Reduced service package - Bedsores - Psychopharmalogical medication - Vulnerable support system - Medication adherence - Dehydration - Heart and lungs - Problem behavior - Palliative care - Alcohol abuse - Risk intramural admission - Physical and mental abuse | - CN conducts CGA, defines, executes and monitors care plan, evaluates changes in care needs and adapt care plan when needed. - CN consults PCP where necessary. - CN leaves copy of the care plan at a person’s home to inform other visiting health professionals and to encourage them to add notes to the care plan. - CN are taught to use an overview of local social and health services including contact details to easily recommend and refer to these services. | - CN takes preferences of older person into consideration in the design and execution of care plan. |
| Kono, 2009 (Kono et al., 2009) | Preventive Home Visit program | - In total, 40 components - CGA includes structured questions - Assessment of locomotion, daily activities, social contacts or relationships with other people and health conditions are suggested by the principles of the Japanese Ministry; authors added inspection for signs of elder abuse. | - CN, CM or SW conducts CGA during home visit. | - Locomotion (e.g. ambulating out of house) - Daily activities (e.g. having meals) - Social contacts or relationships with other people (e.g. going outdoors) - Health conditions (e.g. subjective health status) - Abuse signs (e.g. physical, emotional) - Other | - Home visitor conducts regular home visits to assess care needs, list health and social problems or difficulties, and provide specific recommendations for each older person including attention from community members, consultation from community care professionals, care management or community-based care service, or urgent care; home visitors refer to PCP or CM if older person need medical intervention. | - Home visitor records contribution or comments of older person and informal carer during CGA and gives tailored recommendations based on identified care problems. - Recommendations include providing information on healthcare and social resources, and advising on health care issues and family relationships. - Additional home visits or telephone contacts may be provided to older person in some care need categories. |
| Kono, 2016 (Kono, Izumi, Yoshiyuki, Kanaya, & Rubenstein, 2016); Kono, 2014 (Kono, Izumi, Kanaya, Tsumura, & Rubenstein, 2014)* | Updated Preventive Home Visit Program | - In total, 34 components - CGA is conducted based on systematic structured assessment sheet of care needs - CGA includes internationally validated instruments | - CN, CM or SW - conducts CGA during home visit. | - Face sheet (e.g. socio-demographic characteristics) - Health (e.g. pain) - Mental status (e.g. cognitive function) - Activities (e.g. mobility in house) - Participation (e.g. frequency of going outdoors | - CN, CM or SW conducts regular home visits to do a structured interview, consider demands of older person and identify care needs, demands, assess which steps of preventive care are needed, and initiate comprehensive recommendations including the need of continuous long-term or health care - When necessary, additional home visits or telephone contacts are conducted between visits. | - Preferences of older person and informal carers were assessed - Steps in terms of preventive care include establishing relationships between older person and home visitor |
| Looman, 2014 (Looman, Fabbricotti, & Huijsman, 2014); Looman, 2016 (Looman, Fabbricotti, de Kuyper, & Huijsman, 2016); Fabbricotti, 2013 (Fabbricotti et al., 2013)* | WICM | EASYcare instrument | - CM conducts CGA during home visit. - GP attends executive training in geriatric care, a course in GP consults and EASYcare training. - CM attends EASYcare training and a course in case management. | - (I)ADL - Cognition - Mood - Goal setting | - CM conducts CGA, develops and discusses care plan in multidisciplinary meeting led by GP attended by other health professionals when necessary. - CM is responsible for planning and coordination of care delivery including admittance to required services, monitoring the older person and evaluating care plan. - GP has final responsibility. - WICM is embedded in network structure which consists of GP practices, home care organizations, nursing homes and patient organizations. | - CM consults older people and their caregivers to define treatment goals. - GP harmonizes the care plan with older people and caregivers and obtains permission for its implementation. |
| Mazya, 2013 (Mazya et al., 2013) | AGe-FIT | - In total, 15 components - CGA includes bundle of instruments: instruments (e.g. Memorial, Symptom Assessment Scale), newly developed questionnaire (e.g. feeling of security), questions (e.g. social support) and physical and neurological examination. | - Interdisciplinary team members conduct own part of CGA in several contacts via a telephone call, during a visit at the ambulatory clinic or in the home of the older person, depending on the professional, older person’s condition and ability to travel. - Additional assessments are conducted when considered necessary. | - Medical assessment (e.g. nutritional status) - Assessment of functioning (e.g. walking speed) - Psychological and cognitive assessment (e.g. depression) - Social assessment (e.g. social support) - Overall assessments (e.g. degree of frailty) | - Interdisciplinary team members conduct own part of CGA. - Physician conducts additional assessments, shows outcomes of CGA so far, refer for additional examinations, adjusts medication list and shows plan for next contact. - Interdisciplinary team discuss older person and decide on further action to be taken by team members or by referrals to other care providers, and follow-up. | - Team asks older person to bring a relative or a close friend with them for the physician visit. - The team welcomes next of kin as an important part of the care team and offers them guidance and support. |
| Melis, 2008 (Melis et al., 2008); Melis, 2005 (Melis et al., 2005)*; Richardson, 2001 (Richardson, 2001)* | DGIP | EASYcare instrument   - CGA includes different questions, scales and tests (i.e. short cognitive impairment test), an area for goal setting and monitoring and other information. | - Geriatric specialist nurse conducts CGA during home visit. | - (I)ADL - Cognition - Mood - Goal setting | - PCP refers older person to geriatrician and geriatric specialist nurse conducts CGA. - Intervention team develops care plan and discusses interventions with PCP. - Geriatric specialist nurse conducts main part of intervention, follow up visits for additional geriatric evaluation and management, and consults and advises other involved health care workers. - PCP continues usual care, makes referrals, medication changes, and other interventions as agreed upon during interdisciplinary consultations, continues to be primarily responsible for care and makes final decisions | - GP negotiates a preliminary goal with older person before referring to intervention team, which is further elaborated in an operational objective with geriatric specialist nurse during CGA. |
| Moore, 2012 (Moore et al., 2012) | SCCP | - CGA includes standardized assessments for falls and cognition (based on Quality Indicators for   the Management and Prevention of Falls and the Third  Canadian Consensus Conference on the Diagnosis and Treatment of Dementia), and identification of goals and preferences. | - FP or NP conducts CGA at Family Health Centre or, for housebound older people, in their homes. - Additional assessments by other professionals are conducted when identified needs are beyond expertise of professionals conducting CGA. | - Falls - Cognition - Goals and preferences of older person and informal care | - Registered PN screens for depression, nutrition and medication risks to involve appropriate team members with care. - FP or NP conducts CGA and access other team members when identified needs exceeded the scope of the FP, NP or RPN. - Team members review cases during meetings with other learners and practitioners involved with care, and communicate and implement care plans. - Geriatrician provides consultation during meetings. - Team members, older person’s main care team and community care providers negotiate follow-up arrangements | - FP or NP identifies goals and preferences of older person and informal carer during CGA. - Team members, older person’s main care team and community care providers negotiate plan and follow-up arrangement with older person and informal carer. |
| Parsons, 2013 (Parsons, Sheridan, Rouse, Robinson, & Connolly, 2013); Parsons, 2012 (Parsons, Rouse, Robinson, Sheridan, & Connolly, 2012)* | Model of Restorative Home Care | - CGA includes New Zealand standardized comprehensive geriatric assessment (i.e. Support Needs Assessment), outcome tools (e.g. Nottingham Extended ADL Scale), Goal setting tool (i.e. [TARGET]) | - Assessment staff and homecare coordinators attends a standardised training programme developed for shared understanding of implementation of restorative home-based care and support, and visit peer-review sessions, comprising presentation of completed TARGETs and discussion around implementation of the service delivery plan. | - Cognition, - Informal caregiver stress - Safety - Nutrition - Level of functioning - Health status - Aims of the rehabilitation episode | - Needs assessor conducts CGA and develops goal ladder. - Home care coordinator develops instructions for home care aid in form of support plan, including use of allied health professionals to provide expert guidance in tasks required to attain participant’s goal. | - The needs assessor uses goal-setting tool to identify long-term and short-term goals to form a goal ladder, and home care coordinator developed concrete instructions for the home care aide in the form of a support plan based on goal ladder. |
| Ploeg, 2010 (Ploeg et al., 2010) | Preventive primary care outreach intervention | RAI-HC | - HCN conducts CGA during home visit. | The paper(s) did not describe the domains that were addressed during the CGA. | - HCN conducts CGA, informs FP about outcomes of CGA and actions to be taken by HCN and areas of follow-up by FP. - HCN collaborates with FP and other professionals to implement care plan (e.g. referral to community health and support services), and monitors adherence to recommendations of older person through follow-up phone calls and visits. | - HCN encourages older person to take an active part in their health care and works closely with them and their families. - HCN provides health promotion materials and health education. - HCN refers to various community health and support services in negotiation with older person and informal carers. - HCN leaves card in the home outlining their interventions and any actions required by the older person. |
| Rogerson, 2006 (Rogerson, Weiss, & Phillips, 2006) | GRT | - CGA includes 20-page assessment covering more than 300 questions. - Format is previously developed under the Second Generation Social HMO Demonstration Program. | - A nurse conducts CGA during home visit, which takes about two to three hours. | - Medical - Psychological - Functional - Social - Home safety - Medication | - A member of the GRT team conducts CGA and develops care plan. - GRT team provides PCP with care plan and follow-up conversation when needed. - CM provides direction to older person on how to access community services and implement the care plan. - A member of GRT team conducts follow-up phone calls to evaluate implementation of recommended services. | - GRT team works closely with older person and informal carers to define short-term and long-term goals. - GRT team develops care plan with input from the older person and informal carers. - CM assists older people to implement plan. |
| Rosenberg, 2012 (Rosenberg, 2012) | PIECH | - CGA includes a medical history and examination, functional review, standardized scales (e.g. Mini-Mental State Examination), comprehensive laboratory testing, and discussing healthcare directives. | - PCP and nurse conduct CGA in PIECH practice; PT conducts Berg Balance Scale. - The regional laboratory performs laboratory monitoring, including electrocardiograms, in the home for people who are unable to get out. | The paper(s) did not describe the domains that were addressed during the CGA. | - PCP and nurse conduct CGA and monitor older person during regular home visits, PT conducts own part of CGA, regional laboratory performs laboratory monitoring. - Nurse mobilizes community resources. - Team liaises with older person, informal carers, CMs, CNs and aides, and pharmacists. - CN from regional long-term care program provides long-term procedural services, PCP refers to PT when needed, team recommends home support services when needed, on-call PCP provides after-hours telephone coverage. - Hospitalists and medical specialists provide hospital care; health history is sent to hospital in case of hospitalization; PCP visits hospital to provide supportive care and assist with discharge planning. | - Team aims to allow informed choices about intensity of medical interventions. - Team liaises with older person and informal carers. |
| Ruikes, 2012 (Ruikes et al., 2012); Ruikes, 2016 (Ruikes et al., 2016); van Kempen, 2013 (Van Kempen et al., 2013)* | CareWell | EASYcare-TOS step 2 instrument   - Exact content of CGA is available in appendix of paper - In total, 14 components - EASYcare-TOS step 2 questionnaire is part of the EASYcare assessment system. - Individual patients’ health-related goals and needs on the domains of cure, care and welfare are obtained with the EasyCare-TOS. - EASYcare -TOS has shown good construct validity and interrater reliability, and is tested for its feasibility. | - CN or research assistant conducts CGA during home visit. | - Multimorbidity - Medication - Cognitive problems - Mobility and falling - Looking after yourself - Seeing, hearing and communicating - Staying healthy - Nourishment - Safety - Loneliness/social network - Psychosocial problems - Additional comments - Complexity of the care context | - CN or research assistant conducts CGA. - Multidisciplinary team members review and adapt care plan during team meetings. - CM (CN or gerontological SW, depending on older person’s needs) organizes team meetings, coordinates and monitors care process according to the care plan, as directed by GP. - GP, CN and pharmacist conducts medication review. | - Care plan is formulated based on individual health-related goals and needs. - CM is instructed to ensure older person’s acknowledgement of care plans, encourage involvement in setting goals, and actively maintain treatment contact with older person and their informal carers by home visits and telephone contacts. |
| Schubert, 2016 (Schubert, Myers, Allen, & Counsell, 2016) | GRACE at VAMC | GRACE tool | - GRACE team conducts CGA during home visit. | The paper(s) did not describe the domains that were addressed during the CGA. | - Support team conducts CGA and discuss older person during interdisciplinary team meeting to develop care plan. - Support team collaborates with PCP to review, modify, and implement care plan. - Support team performs follow-up visits. | - Support team visits the older person to build rapport with the older person before conducting CGA - Support team collaborates with older person to review, modify, and implement care plan in a way consistent with older person’s goal of care - Support team continued to participate in care collaboratively through monthly and as-needed follow-up visits, face-to-ace or over the telephone, as clinically indicated. |
| Spoorenberg, 2013 (Spoorenberg et al., 2013); Spoorenberg, 2018 (Spoorenberg, Wynia, Uittenbroek, Kremer, & Reijneveld, 2018); Uittenbroek, 2016 (Uittenbroek, Kremer, Spoorenberg, Reijneveld, & Wynia, 2016); Spoorenberg, 2015 (Spoorenberg et al., 2015)* | Embrace | Geriatric ICF Core Set   - In total, 30 components - CGA is a structured history questionnaire - Instrument is based on the results of a Delphi study. - Categories are selected from the International Classification of Functioning, Disability and Health (ICF). - Severity of the problems identified estimated using severity scores ranging from 0 (no problem) to 10 (complete problem). | - CM provides assistance in filling out this form during home visit. - Additional examinations are conducted in case of multimorbidity. - ECT members follow an intensive training program that focuses on working according to Embrace, for example, pro-active teamwork, prevention: district nurse or SW to fulfil role as CM and to perform self-management interventions, GP to manage teams and provide care and support targeting specific problems. In addition, ECT members were coached to support cultural change in working patterns during intervention. | - Body functions (e.g. hearing functions, memory functions) - Activities and participation (e.g. changing basic body position, using transportation) - Environmental factors (e.g. immediate family, friends) | - Intensity, focus and approach of care and support depend on risk profile of older person - For frail older people and people with complex needs, CM (district nurse or SW, depending on older person’s needs) conducts CGA, and GP or elderly care physician conducts additional examinations. - CM formulates care and support plan and puts it into practice after consultation with Elderly Care Team led by GP. - CM monitors status of the older person and implementation of care plan, and keeps in contact with involved professionals and volunteers being employed. - Elderly Care Team discusses progress in reaching goals and effectiveness of interventions. - CM navigate older person through complex processes of organizing care and support. - GP and elderly care physician manage the medical care of older people with multimorbidity. | - CM formulates a care and support plan in consultation with the older person, based on identified health problems relevant to the older person. For each health problem, goal scores are estimated and suitable actions selected. - Care plan put into practice after final approval by the older person. |
| De Stampa, 2014 (De Stampa et al., 2014); Vedel, 2009 (Vedel et al., 2009); Morris, 1997 (Morris et al., 1997)* | COPA | RAI-HC   - RAI-HC is developed by InterRAI; validity and reliability of RAI-HC has been well documented. | - CM conducts CGA during home visit. | - Personal items - Cognitive patterns - Communication/hearing - Vision - Mood and behavior - Social functioning - Informal support services - Physical functioning - Continence - Disease diagnoses - Health conditions and preventive health measures - Nutrition/hydration - Dental status - Skin condition - Environmental assessment - Service utilization - Medications | - Nurse conducts CGA, consults multidisciplinary team to develop care plan, discusses care plan with PCP, implement care plan and coordinates health and social services across different settings and care providers including inpatient visits and hospital discharge with hospital team, follows up on and reassesses needs of older person and is in contact with PCP to consult on complicated cases. - PCP participates in care management, is responsible for medical decision making and makes referrals to medical specialists including geriatricians. - Two-person team is responsible for older person’s care trajectory. - Geriatrician provides support to two-person team or PCPs and coordinates relationships between hospitalist physicians and PCPs including hospital admissions. - Psychologist provides socio-psychological support for older person and their family. | - CM consults multidisciplinary team to develop care plan that meets older person’s expectations, available resources and needs. - Older person and informal carer can reach CM if a problem arises. |
| Stijnen, 2013 (Stijnen, Duimel-Peeters, Jansen, & Vrijhoef, 2013); Stijnen, 2013 (Stijnen, Jansen, Vrijhoef, & Duimel-Peeters, 2013)* | [G]OLD | [G]OLD instrument   - Exact content of CGA is available in paper - In total, 23 components in basic assessment and 6 components in additional examination - CGA includes bundle of instruments: (items from) validated instruments (e.g. Mini Mental State Examination), slightly adjusted validated tests (e.g. IADL scale), questions formulated by expert panel based on clincial expertise or derived from clinical guidelines (e.g. performing outdoor activities,), and biomedical measurement (e.g. blood pressure). - Content is based on an existing Dutch instrument for geriatric assessment TRAZAG and on input from expert panel. - CGA is specifically developed for and tested among target population in a pilot study. - Instrument was tested for practical usefulness by PNs and GPs. | - PN conducts CGA during home visit. - PN makes a print out of the person’s medication list and medical history for relevant details or major events to be aware of, before home visit. - Additional examinations are conducted if more insight is needed. - PN receives two days of training before intervention provide them with the necessary knowledge and skills including conducting CGA and communication skills, etc.; sessions are organised for asking questions and exchanging experiences during intervention; PN received additional support by a coach specialised in geriatric care. - Home visits took on average 55.7 minutes. | - Physical functioning (e.g. incontinence) - Psychological functioning (e.g. cognition) - Mental functioning - Social functioning (e.g. social participation) - Lifestyle (e.g. smoking) - Medication use | - PN conducts CGA and perform additional examination. - PN discusses outcomes of CGA with GP and (together) formulates care and treatment plan. - PN arranges and coordinates care for the older person including additional diagnosis, preventive care or advice, treatment in primary health care and referral to other care and/or well-being facilities, and monitors progress and follow-up taking place with PN or any of the other care providers to whom older people are being referred. | - PN establishes a relationship of trust, listens to the needs and wishes of the older person, allows them time to talk, and provides information or advice if necessary, during CGA. - PN discusses results of CGA with the older person, and PN (and/or GP) develops draft care plan in accordance with older person’s needs and wishes, and discuss this with older person, whose input and wishes lead to final care and treatment plan. - PN, GP and older person discusses how to proceed if follow-up actions are not required or desirable from patient’s point of view. |
| Suijker, 2012 (Suijker et al., 2012); Suijker, 2016 (Suijker et al., 2016) | FIT | CGA FIT   - Full content of CGA is available in paper - In total, 27 components - CGA includes bundle of instruments: internationally validated instruments, commonly used items from guidelines or literature, and physical examination - Some components are used for all older people; some are used following a positive answer on a screening question. - Instrument is based on experience from a previous study by an expert panel and supported by older volunteers, and its feasibility was tested during pilot phase of study. | - RN conducts CGA during two home visits which take about 40 to 60 minutes. - RN follow a 10-day training program on conducting CGA and designing and applying tailored care and treatment plan including care coordination, patient empowerment and motivational interviewing, and attend regular group refresher courses to discuss complex cases, etc. | - Physical domain (e.g mobility and stability) - Psychological domain (e.g. cognition) - Functional domain (e.g. ADL functioning) - Social domain (e.g. loneliness) - Physical examination (e.g. walking speed) | - RN conducts CGA and further diagnostic assessments, and collaborates with GP to discuss outcomes of CGA and to develop care and treatment plan in which all actions expected of the older person, RN and GP are specified. Possible interventions are referral to other professionals, giving advice or a follow-up visit. - RN conducts follow-up visits to evaluate care plan and the need for continuation of care coordination, and is responsible for care coordination and collaborates with GP and maintains contacts with other health care professionals and informal carers. - GP has final responsibility for care and treatment. | - RN asks older person whether they recognized identified conditions as problems and desired (additional) care or treatment for them, and to prioritize problems during CGA. - RN discusses care plan with older person and their caregivers, in which all actions expected of older person are specified; potential discrepancies in priorities between older person, RN and GP are addressed. - RN discusses several themes during subsequent home visits, including prioritizing identified conditions, needs and expectations, etc. - RN enhances empowerment of older person and informal carers by providing or facilitating psychoeducation. - Informal carer is invited to enhance older person’s adherence to intervention. |
| Tracy, 2013 (Tracy, Bell, Nickell, Charles, & Upshur, 2013) | IMPACT | - CGA includes quality-of-life interview and interprofessional assessments | - Comprehensive team conducts CGA during visit at IMPACT clinic, which takes about one and a half to two hours. - Visit consists of three interprofessional discussion rounds: discussing priorities and concerns of older person and informal carers to define sequence of assessments during visit; discussing findings and drafting care plan; and discussing follow-up plan. - During assessments rest of the team observes via closed-circuit television. - Older person is encouraged to bring family members or paid caregivers, their current medication and a list of current concerns to their appointment. | - Quality-of-life: patient’s circumstances and concerns - Medication - Mobility - Nutrition - Mobility - Cognition - Home safety - Sensory - Nursing issues | - FP resident conducts quality-of-life interview - Each team member conducts assessment related to their own discipline and observes each other’s assessments. - Team develops care plan and arranges referrals and follow-up appointments. - Team develops follow-up care plan for FP who resumes ongoing care. | - Older person is encouraged to bring family member or paid caregiver involved in care to the appointment. - FP residents conducts quality-of-life interview to unpack circumstances and concerns of older person. - Team discusses priorities of older person and informal carer to define sequence of assessments. - Team provides information package including patient-friendly to-do list, medication list, other educational information and resources, and referrals and follow-up appointments. - FP and resident review the care plan and the information package with older person and informal carer, and encourages them to raise concerns, ask questions, and discuss the specifics of the care plan. |

*: added after reference tracking; ADL: activities of daily living; CGA: Comprehensive Geriatric Assessment; CM: case manager; CN: community nurse; FP: family physician; GP: general practitioner; HCN: home care nurse; IADL: instrumental activities of daily living; NP: nurse practitioner; OT: occupational therapist; PCP: primary care physician; PN: practice nurse; PT: physical therapist RN: registered nurse; SW: social worker.

Blom, J., Den Elzen, W., Van Houwelingen, A. H., Heijmans, M., Stijnen, T., Van den Hout, W., & Gussekloo, J. (2016). Effectiveness and cost-effectiveness of a proactive, goal-oriented, integrated care model in general practice for older people. A cluster randomised controlled trial: Integrated Systematic Care for older People—the ISCOPE study. *Age and Ageing, 45*(1), 30-41. doi:10.1093/ageing/afv174

Boult, C., Leff, B., Boyd, C. M., Wolff, J. L., Marsteller, J. A., Frick, K. D., . . . Scharfstein, D. O. (2013). A matched-pair cluster-randomized trial of Guided Care for high-risk older patients. *Journal of General Internal Medicine, 28*(5), 612-621. doi:10.1007/s11606-012-2287-y

Bouman, A., Van Rossum, E., Ambergen, T., Kempen, G., & Knipschild, P. (2008). Effects of a home visiting program for older people with poor health status: a randomized, clinical trial in the Netherlands. *J Am Geriatr Soc, 56*(3), 397-404. doi:10.1111/j.1532-5415.2007.01565.x

Boyd, C. M., Boult, C., Shadmi, E., Leff, B., Brager, R., Dunbar, L., . . . Wegener, S. (2007). Guided Care for multimorbid older adults. *Gerontologist, 47*(5), 697-704. doi:10.1093/geront/47.5.697

Boyd, C. M., Shadmi, E., Conwell, L. J., Griswold, M., Leff, B., Brager, R., . . . Boult, C. (2008). A pilot test of the effect of guided care on the quality of primary care experiences for multimorbid older adults. *Journal of General Internal Medicine, 23*(5), 536-542.

Brettschneider, C., Luck, T., Fleischer, S., Roling, G., Beutner, K., Luppa, M., . . . König, H. H. (2015). Cost-utility analysis of a preventive home visit program for older adults in Germany. *BMC Health Services Research, 15*(1), 141. doi:10.1186/s12913-015-0817-0

Buurman, B. M., Parlevliet, J. L., Allore, H. G., Blok, W., Van Deelen, B. A. J., Moll Van Charante, E. P., . . . De Rooij, S. E. (2016). Comprehensive geriatric assessment and transitional care in acutely hospitalized patients the transitional care bridge randomized clinical trial. *JAMA Internal Medicine, 176*(3), 302-309. doi:10.1001/jamainternmed.2015.8042.

Buurman, B. M., Parlevliet, J. L., van Deelen, B. A., de Haan, R. J., & de Rooij, S. E. (2010). A randomised clinical trial on a comprehensive geriatric assessment and intensive home follow-up after hospital discharge: the Transitional Care Bridge. *BMC Health Services Research, 10*, 296.

Counsell, S. R., Callahan, C. M., Buttar, A. B., Clark, D. O., & Frank, K. I. (2006). Geriatric Resources for Assessment and Care of Elders (GRACE): a new model of primary care for low-income seniors. *J Am Geriatr Soc, 54*(7), 1136-1141.

Counsell, S. R., Callahan, C. M., Clark, D. O., Tu, W., Buttar, A. B., & Stump, T. E. (2007). Geriatric care management for low-income seniors: a randomized controlled trial. *JAMA, 298*. doi:10.1001/jama.298.22.2623

Daniels, R., van Rossum, E., Metzelthin, S., Sipers, W., Habets, H., Hobma, S., . . . de Witte, L. (2011). A disability prevention programme for community-dwelling frail older persons. *Clinical Rehabilitation, 25*(11), 963-974.

De Stampa, M., Vedel, I., Buyck, J. F., Lapointe, L., Bergman, H., Beland, F., & Ankri, J. (2014). Impact on hospital admissions of an integrated primary care model for very frail elderly patients. *Archives of Gerontology and Geriatrics, 58*(3), 350-355. doi:http://dx.doi.org/10.1016/j.archger.2014.01.005

Fabbricotti, I. N., Janse, B., Looman, W. M., de Kuijper, R., van Wijngaarden, J. D. H., & Reiffers, A. (2013). Integrated care for frail elderly compared to usual care: a study protocol of a quasi-experiment on the effects on the frail elderly, their caregivers, health professionals and health care costs. *BMC Geriatrics, 13*(1), 31. doi:10.1186/1471-2318-13-31

Faul, A. C., Yankeelov, P. A., Rowan, N. L., Gillette, P., Nicholas, L. D., Borders, K. W., . . . Wiegand, M. (2009). Impact of geriatric assessment and self-management support on community-dwelling older adults with chronic illnesses. *Journal of Gerontological Social Work, 52*(3), 230-249.

Fleischer, S., Roling, G., Beutner, K., Hanns, S., Behrens, J., Luck, T., . . . Lautenschläger, C. (2008). Growing old at home – A randomized controlled trial to investigate the effectiveness and cost-effectiveness of preventive home visits to reduce nursing home admissions: study protocol [NCT00644826]. *BMC Public Health, 8*, 185. doi:10.1186/1471-2458-8-185

Hertogh, C. M. P. M., Deerenberg-Kessler, W., & Ribbe, M. W. (1996). The problem-oriented multidisciplinary approach in Dutch nursing home care. *Clinical Rehabilitation, 10*(2), 135-142. doi:10.1177/026921559601000209

Hoogendijk, E. O., Van Der Horst, H. E., Van De Ven, P. M., Twisk, J. W. R., Deeg, D. J. H., Frijters, D. H. M., . . . Van Hout, H. P. J. (2016). Effectiveness of a Geriatric Care Model for frail older adults in primary care: results from a stepped wedge cluster randomized trial. *European Journal of Internal Medicine, 28*, 43-51. doi:10.1016/j.ejim.2015.10.023

Kono, A., Fujita, T., Tsumura, C., Kondo, T., Kushiyama, K., & Rubenstein, L. Z. (2009). Preventive home visit model targeted to specific care needs of ambulatory frail elders: Preliminary report of a randomized trial design. *Aging Clinical and Experimental Research, 21*(2), 167-173.

Kono, A., Izumi, K., Kanaya, Y., Tsumura, C., & Rubenstein, L. Z. (2014). Assessing the quality and effectiveness of an updated preventive home visit programme for ambulatory frail older Japanese people: research protocol for a randomized controlled trial. *Journal of advanced nursing, 70*(10), 2363-2372.

Kono, A., Izumi, K., Yoshiyuki, N., Kanaya, Y., & Rubenstein, L. Z. (2016). Effects of an Updated Preventive Home Visit Program based on a systematic structured assessment of care needs for ambulatory frail older adults in Japan: A randomized controlled trial. *The journals of gerontology. Series A, Biological sciences and medical sciences, 71*(12), 1631-1637. doi:10.1093/gerona/glw068

Looman, W. M., Fabbricotti, I. N., de Kuyper, R., & Huijsman, R. (2016). The effects of a pro-active integrated care intervention for frail community-dwelling older people: a quasi-experimental study with the GP-practice as single entry point. *BMC Geriatrics, 16*, 43. doi:http://dx.doi.org/10.1186/s12877-016-0214-5

Looman, W. M., Fabbricotti, I. N., & Huijsman, R. (2014). The short-term effects of an integrated care model for the frail elderly on health, quality of life, health care use and satisfaction with care. *International Journal of Integrated Care, 14*(4).

Mazya, A. L., Eckerblad, J., Jaarsma, T., Hellström, I., Krevers, B., Milberg, A., . . . Ekdahl, A. (2013). The Ambulatory Geriatric Assessment - A Frailty Intervention Trial (AGe-FIT) - A randomised controlled trial aimed to prevent hospital readmissions and functional deterioration in high risk older adults: A study protocol. *European Geriatric Medicine, 4*(4), 242-247.

Melis, R. J. F., Van Eijken, M. I. J., Borm, G. F., Wensing, M., Adang, E., Van de Lisdonk, E. H., . . . Olde Rikkert, M. G. M. (2005). The design of the Dutch EASYcare study: a randomised controlled trial on the effectiveness of a problem-based community intervention model for frail elderly people [NCT00105378]. *BMC Health Services Research, 5*(1), 65. doi:10.1186/1472-6963-5-65

Melis, R. J. F., Van Eijken, M. I. J., Teerenstra, S., Van Achterberg, T., Parker, S. G., Borm, G. F., . . . Olde Rikkert, M. G. M. (2008). A randomized study of a multidisciplinary program to intervene on geriatric syndromes in vulnerable older people who live at home (Dutch EASYcare Study). *The journals of gerontology. Series A, Biological sciences and medical sciences, 63*(3), 283-290.

Metzelthin, S. F., Van Rossum, E., De Witte, L. P., Ambergen, A. W., Hobma, S. O., Sipers, W., & Kempen, G. I. J. M. (2013). Effectiveness of interdisciplinary primary care approach to reduce disability in community dwelling frail older people: cluster randomised controlled trial. *British Medical Journal, 347*(7926).

Metzelthin, S. F., van Rossum, E., de Witte, L. P., Hendriks, M. R., & Kempen, G. I. (2010). The reduction of disability in community-dwelling frail older people: design of a two-arm cluster randomized controlled trial. *BMC Public Health, 10*, 511. doi:10.1186/1471-2458-10-511

Moore, A., Patterson, C., White, J., House, S. T., Riva, J. J., Nair, K., . . . McCann, D. (2012). Interprofessional and integrated care of the elderly in a family health team. *Canadian Family Physician, 58*(8), e436-e441.

Morris, J. N., Fries, B. E., Steel, K., Ikegami, N., Bernabei, R., Carpenter, G. I., . . . Topinková, E. (1997). Comprehensive Clinical Assessment in Community Setting: Applicability of the MDS-HC. *J Am Geriatr Soc, 45*(8), 1017-1024. doi:10.1111/j.1532-5415.1997.tb02975.x

Muntinga, M. E., Hoogendijk, E. O., van Leeuwen, K. M., van Hout, H. P. J., Twisk, J. W. R., Van der Horst, H. E., . . . Jansen, A. P. D. (2012). Implementing the chronic care model for frail older adults in the Netherlands: study protocol of ACT (frail older adults: care in transition). *BMC Geriatrics, 12*(1), 1-10. doi:10.1186/1471-2318-12-19

Nicolaides-Bouman A, van Rossum E, Kempen GIJM, & Knipschild P. (2004). Effects of home visits by home nurses to elderly people with health problems: design of a randomised clinical trial in the Netherlands [ISRCTN92017183]. *BMC Health Serv Res, 4*, 35. doi:10.1186/1472-6963-4-35

Nicolaides-Bouman, A., van Rossum, E., Kempen, G. I. J. M., & Knipschild, P. (2004). Effects of home visits by home nurses to elderly people with health problems: design of a randomised clinical trial in the Netherlands [ISRCTN92017183]. *BMC Health Services Research, 4*, 35. doi:10.1186/1472-6963-4-35

Parsons, J., Rouse, P., Robinson, E. M., Sheridan, N., & Connolly, M. J. (2012). Goal setting as a feature of homecare services for older people: does it make a difference? *Age & Ageing, 41*(1), 24-29. doi:10.1093/ageing/afr118

Parsons, J. G. M., Sheridan, N., Rouse, P., Robinson, E., & Connolly, M. (2013). A randomized controlled trial to determine the effect of a model of restorative home care on physical function and social support among older people. *Archives of Physical Medicine and Rehabilitation, 94*(6), 1015-1022. doi:10.1016/j.apmr.2013.02.003

Ploeg, J., Brazil, K., Hutchison, B., Kaczorowski, J., Dalby, D. M., Goldsmith, C. H., & Furlong, W. (2010). Effect of preventive primary care outreach on health related quality of life among older adults at risk of functional decline: randomised controlled trial. *BMJ, 340*, c1480. doi:http://dx.doi.org/10.1136/bmj.c1480

Richardson, J. (2001). The Easy-Care assessment system and its appropriateness for older people. *Nursing Older People, 13*(7), 17-19. doi:10.7748/nop.13.7.17.s15

Rogerson, S. L., Weiss, L. J., & Phillips, S. L. (2006). What do our seniors need? Outcomes of at-home comprehensive geriatric assessments. *Annals of Long-Term Care, 14*(10), 31-34.

Rosenberg, T. (2012). Acute hospital use, nursing home placement, and mortality in a frail community-dwelling cohort managed with Primary Integrated Interdisciplinary Elder Care at Home. *J Am Geriatr Soc, 60*(7), 1340-1346. doi:http://dx.doi.org/10.1111/j.1532-5415.2012.03965.x

Ruikes, F. G., Meys, A. R., Van de Wetering, G., Akkermans, R. P., Van Gaal, B. G., Zuidema, S. U., . . . Koopmans, R. T. (2012). The CareWell-primary care program: design of a cluster controlled trial and process evaluation of a complex intervention targeting community-dwelling frail elderly. *BMC Family Practice, 13*, 115.

Ruikes, F. G. H., Zuidema, S. U., Akkermans, R. P., Assendelft, W. J. J., Schers, H. J., & Koopmans, R. T. C. M. (2016). Multicomponent program to reduce functional decline in frail elderly people: A cluster controlled trial. *Journal of the American Board of Family Medicine, 29*(2), 209-217. doi:10.3122/jabfm.2016.02.150214

Schubert, C. C., Myers, L. J., Allen, K., & Counsell, S. R. (2016). Implementing geriatric resources for assessment and care of elders team care in a Veterans Affairs Medical Center: Lessons learned and effects observed. *J Am Geriatr Soc, 64*(7), 1503-1509.

Spoorenberg, S. L., Uittenbroek, R. J., Middel, B., Kremer, B. P., Reijneveld, S. A., & Wynia, K. (2013). Embrace, a model for integrated elderly care: study protocol of a randomized controlled trial on the effectiveness regarding patient outcomes, service use, costs, and quality of care. *BMC Geriatrics, 13*, 62.

Spoorenberg, S. L. W., Reijneveld, S. A., Middel, B., Uittenbroek, R. J., Kremer, H. P. H., & Wynia, K. (2015). The Geriatric ICF Core Set reflecting health-related problems in community-living older adults aged 75 years and older without dementia: development and validation. *Disability and Rehabilitation, 37*(25), 2337-2343. doi:10.3109/09638288.2015.1024337

Spoorenberg, S. L. W., Wynia, K., Uittenbroek, R. J., Kremer, H. P. H., & Reijneveld, S. A. (2018). Effects of a population-based, person-centred and integrated care service on health, wellbeing and self-management of community-living older adults: a randomised controlled trial on Embrace. *PLOS ONE, 13*(1), e0190751. doi:10.1371/journal.pone.0190751

Stijnen, M. M., Duimel-Peeters, I. G., Jansen, M. W., & Vrijhoef, H. J. (2013). Early detection of health problems in potentially frail community-dwelling older people by general practices--project [G]OLD: design of a longitudinal, quasi-experimental study. *BMC Geriatrics, 13*, 7.

Stijnen, M. M. N., Jansen, M. W. J., Vrijhoef, H. J. M., & Duimel-Peeters, I. G. P. (2013). Development of a home visitation programme for the early detection of health problems in potentially frail community-dwelling older people by general practices. *European Journal of Ageing, 10*(1), 49-60. doi:10.1007/s10433-012-0251-7

Suijker, J. J., Buurman, B. M., Ter Riet, G., Van Rijn, M., De Haan, R. J., De Rooij, S. E., & Moll van Charante, E. P. (2012). Comprehensive geriatric assessment, multifactorial interventions and nurse-led care coordination to prevent functional decline in community-dwelling older persons: protocol of a cluster randomized trial. *BMC Health Services Research, 12*, 85.

Suijker, J. J., Van Rijn, M., Buurman, B. M., Ter Riet, G. T., Moll Van Charante, E. P., & De Rooij, S. E. (2016). Effects of nurse-led multifactorial care to prevent disability in community-living older people: Cluster randomized trial. *PLOS ONE, 11*(7).

Tracy, C. S., Bell, S. H., Nickell, L. A., Charles, J., & Upshur, R. E. (2013). The IMPACT clinic: innovative model of interprofessional primary care for elderly patients with complex health care needs. *Canadian Family Physician, 59*(3), e148-155.

Uittenbroek, R. J., Kremer, H. P. H., Spoorenberg, S. W. L., Reijneveld, S. A., & Wynia, K. (2016). Integrated Care for Older Adults Improves Perceived Quality of Care: Results of a Randomized Controlled Trial of Embrace. *Journal of General Internal Medicine, 5*(32), 516-523.

Van Hout, H. P., Jansen, A. P., Van Marwijk, H. W., Pronk, M., Frijters, D. F., & Nijpels, G. (2010). Prevention of adverse health trajectories in a vulnerable elderly population through nurse home visits: a randomized controlled trial [ISRCTN05358495]. *The journals of gerontology. Series A, Biological sciences and medical sciences, 65*(7), 734-742. doi:http://dx.doi.org/10.1093/gerona/glq037

Van Kempen, J. A. L., Schers, H. J., Jacobs, A., Zuidema, S. U., Ruikes, F., Robben, S. H. M., . . . Olde Rikkert, M. G. M. (2013). Development of an instrument for the identification of frail older people as a target population for integrated care. *British Journal of General Practice, 63*(608), e225-e231. doi:10.3399/bjgp13X664289

Vedel, I., De Stampa, M., Bergman, H., Ankri, J., Cassou, B., Mauriat, C., . . . Lapointe, L. (2009). A novel model of integrated care for the elderly: COPA, coordination of professional care for the elderly. *Aging Clinical and Experimental Research, 21*(6), 414-423.
